# Applications of Lignin‐Dervied Carbon Quantum Dots: Current Status and Challenges

**DOI:** 10.1002/EXP.70039

**Published:** 2025-04-17

**Authors:** Xiuxin Yin, Zhili Zhang, Fengfeng Li, Maoqing Fu, Tianci Qin, Xingxiang Ji, Yuanyuan Wang, Zhiwen Wang, Shaolong Sun

**Affiliations:** ^1^ State Key Laboratory of Green Papermaking and Resource Recycling Qilu University of Technology Shandong Academy of Sciences Jinan P. R. China; ^2^ Department of Chemistry Organic and Bioorganic Chemistry University of Graz, Heinrichstrasse Graz Austria; ^3^ College of Natural Resources and Environment South China Agricultural University Guangzhou Guangdong P. R. China

**Keywords:** application, fluorescence properties, LCQDs, lignin, preparation

## Abstract

In recent years, lignin has attracted substantial attention from researchers because of its diverse sources, low cost, and renewability. The effective functionalization and enhanced value‐added utilization of lignin have successfully addressed the challenges associated with biomass resource waste, low utilization rate, high material cost, and underwhelming performance in energy, environmental protection, and medical applications. The emergence of lignin carbon quantum dots (LCQDs) has opened new avenues for the development and utilization of lignin by offering exciting opportunities for their applications. LCQDs possess unique characteristics such as fluorescence properties, size effect, surface effect, and interface effects, which are promising for applications in many fields. This paper provides a comprehensive overview of the structure and applications of lignin with a specific focus on the preparation method of LCQDs as well as their various applications in drug delivery systems, electrode material fabrication, and antibacterial agent development. Furthermore, this study offers valuable insights into the prospects of LCQDs and aims to contribute to their functional development. Finally, the challenges associated with leveraging the fluorescence properties of LCQDs are discussed, along with potential directions for future research.

## Graphical abstract

Based on previous studies, this paper reviews the current preparation methods of lignin carbon quantum dots (LCQDs), and focuses on the preparation of lignin derivatives and various lignin based CQDs fluorescent materials and their applications in heavy metal ion detection, biological imaging, anti‐counterfeiting printing, optical devices and other fields. Finally, the preparation methods of LCQDs and the challenges, research and selection of fluorescent materials for future applications are discussed in detail.

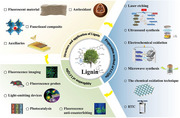

## 1 Introduction

Lignin, a primary constituent of natural wood fibers, is the most prevalent naturally occurring aromatic polymer [1]. Due to the presence of a benzene ring, lignin shares several characteristics and advantages with aromatic compounds [2]. Various functional groups, including carboxyl, methoxy, carbonyl, and other hydroxyl groups, can be found in lignin [3] contributing to its exceptional properties such as antibacterial activity, anti‐ultraviolet protection, antioxidant capacity, and adsorption capability [4].

In recent years, noteworthy progress has been made in the research on lignin carbon quantum dots (LCQDs), mainly focusing on the use of their abundant renewable properties to create high‐value materials [5]. Lignin, a raw biomass material, has multiple advantages in the preparation of CQDs [6]. More meaningfully, the environmental friendliness of LCQDs compared to traditional quantum dots, which typically contain toxic heavy metals, makes them attractive option for sustainable technological development. First, lignin is rich in aromatic rings and carbon elements, and as a precursor of CQDs, it has a unique chemical structure that is conducive to the formation of quantum dots with excellent performance [7]. Second, the preparation of lignin‐based quantum dots can effectively reduce the cost of CQDs production and realize the high‐value waste utilization [8]. The environmental friendliness and potentially high luminescence quantum yield of LCQDs also contribute to the development of green and sustainable technologies. In addition, the size and shape of the LCQDs can be precisely controlled by various synthetic methods, thereby regulating their optical and electronic properties of LCQDs [9]. This provides a broad research space for the application of LCQDs in optoelectronics, sensors, biological imaging and other fields [10]. Finally, LCQDs can accelerate charge transfer and effectively separate photogenerated electrons through their internal electric fields [11]. The excellent activity of LCQDs in photocatalytic applications further expands its potential impact in the field of advanced materials science [12].

In this study, the structure, properties and applications of lignin as an antioxidants, auxiliary, and functional composites are reviewed. The preparation method, properties and applications of LCQDs were mainly introduced, laying a foundation for the high‐value application of lignin and the further development of LCQDs.

## 2 The Structural Properties and Applications of Lignin

**FIGURE 1:**
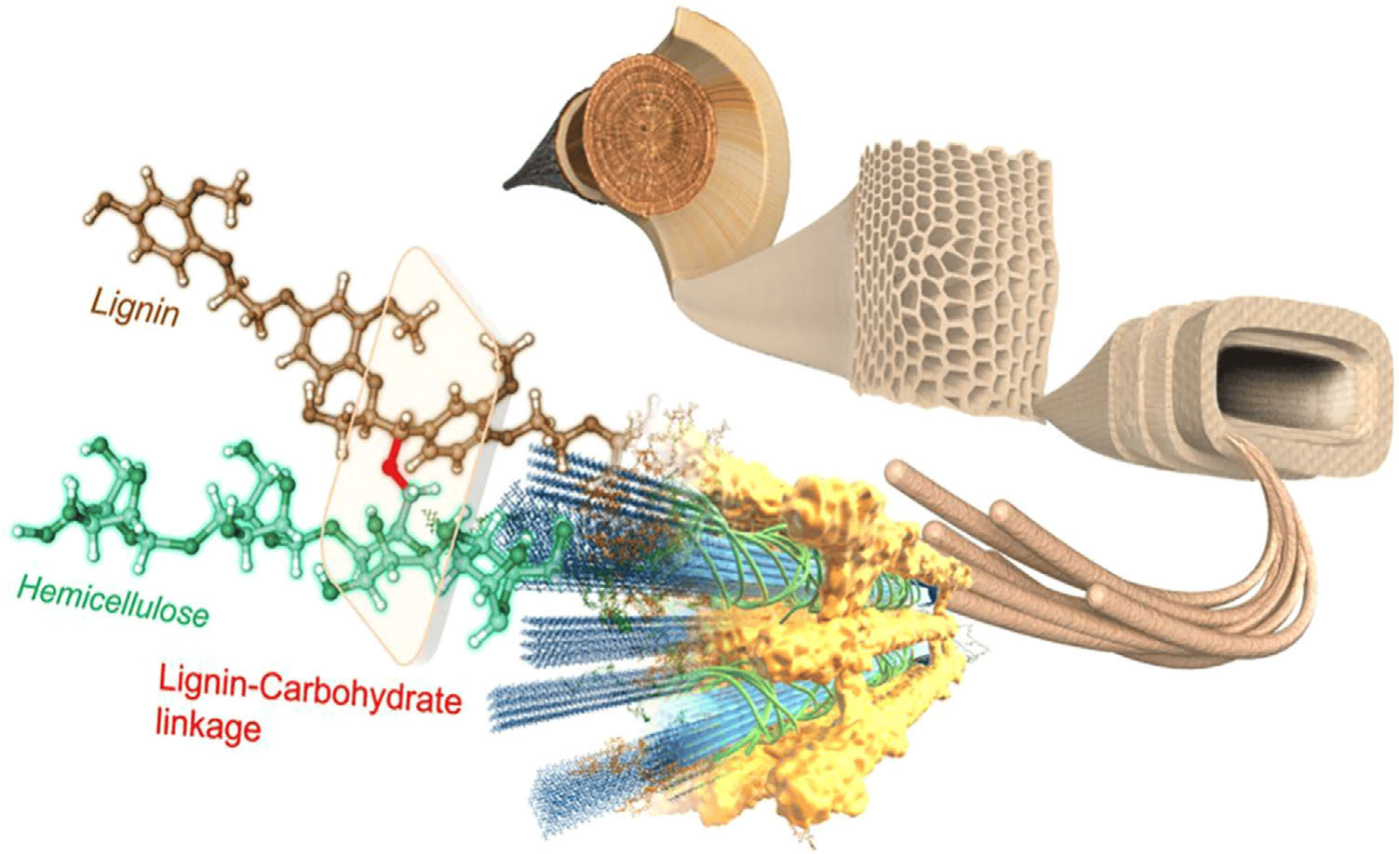
Schematic representation of the arrangement of lignin, cellulose, and hemicellulose in plant cells [18]. Reproduced with permission of Ref. Copyright 2020, MDPI.

Lignocellulose, which is found in large quantities in nature, accounts for approximately 50% of the total annual biomass production [13]. Cellulose, hemicellulose, and lignin are the main components of lignocellulosic biomass and comprise cellulose (40–45%), hemicelluloses (25–35%), and lignin (15–30%) [14]. The arrangement of lignin, cellulose, and hemicellulose in plant cells is shown in Figure 1. Lignin is an aromatic compound with the natural aromatic hydrocarbon structure of complex reticulated biomolecules. Furthermore, lignocellulosic biomass is the second largest naturally occurring renewable resource, surpassed by cellulose [15]. Every year, the paper and pulp industries generate an excess of 50 million tons of waste lignin, but only approximately 5% is used for high‐value utilization, and more than 98% is used as fuel for combustion treatment, resulting in a large amount of greenhouse gas emissions [16]. Therefore, the development of an inexpensive and efficient technology to convert industrial lignin into high‐value‐added functional materials instead of low‐value combustion can reduce environmental pollution caused by combustion. In addition, the realization of the high‐value utilization of industrial waste could meet the needs of low‐carbon social development and have great implication for environmental protection [17]. Hence, it remains crucial to explore avenues for maximizing the value of lignin through ongoing research efforts.

### 2.1 Lignin Structure

**FIGURE 2:**
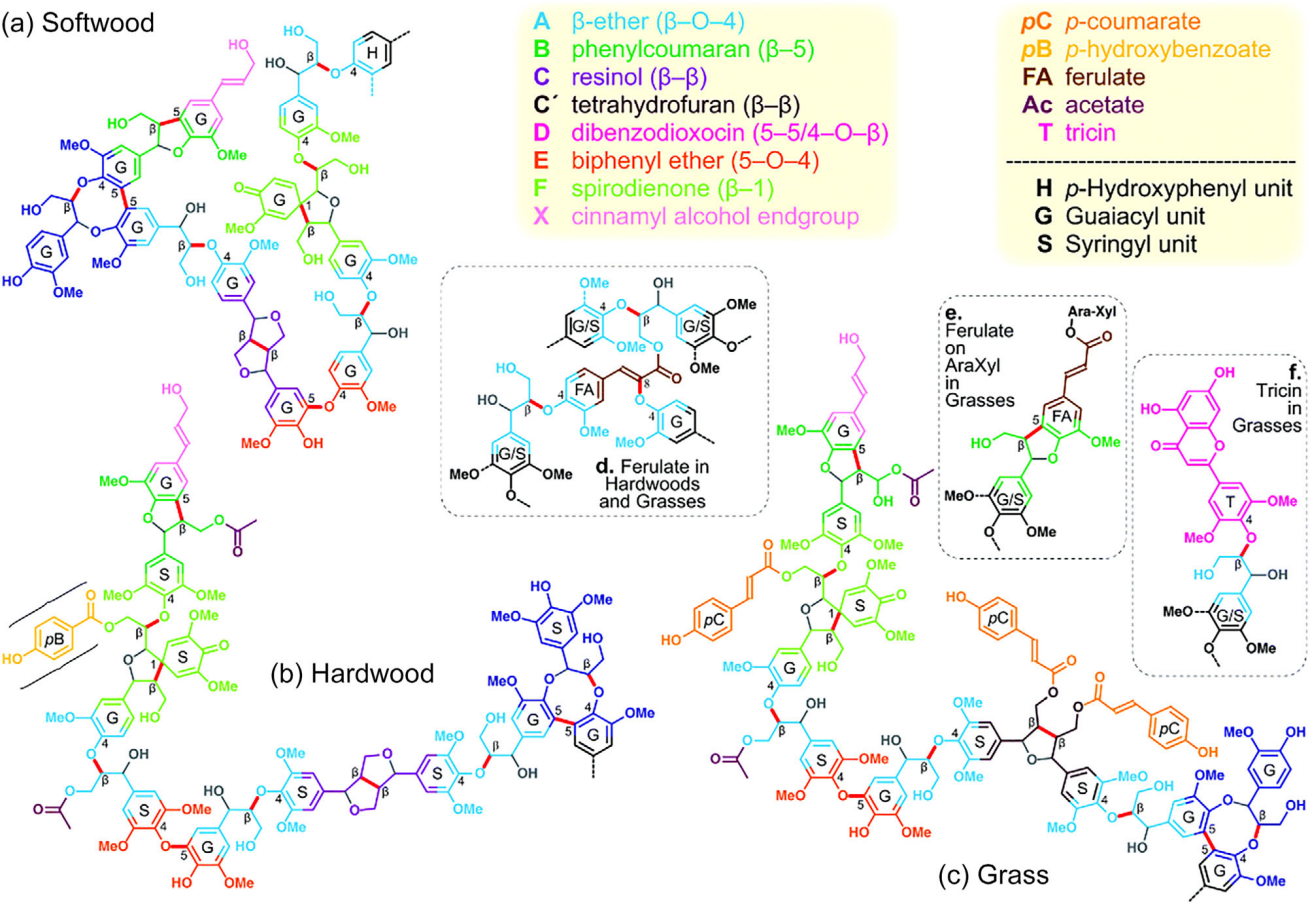
Structural models of lignin in softwood, hardwood and grass materials [25]. Reproduced with permission of Ref. Copyright 2019, Elsevier.

Lignin has a natural aromatic structure and is a heterogeneous, amorphous, and complex natural polymer [18]. Lignin is a sophisticated biomolecular network that primarily consists of three structural units for the polymerization G‐type, S‐type‐, and H‐type polymers [19]. Depending on the source, lignin is typically categorized into three primary groups: softwood, hardwood, and herbaceous. Softwood lignin is mainly derived from coniferous wood such as fir and pine. The primary structural units found in softwood lignin are predominantly G‐type. Hardwood lignin mainly originates from birch and aspen. Its structural units are prioritized over the G‐type and S‐type [20]. Herbaceous lignin is mainly derived from grass and bamboo. Its structural units are primarily composed of G‐, S‐types‐, and H‐types. The primary distinction is that structural separation is induced by varying alterations [21]. In addition, the composition of lignin composition varies across different parts of the plant body. Therefore, lignin structural units may contain many active functional groups, such as methoxy (─OCH3), phenolic hydroxyl (Ph─OH), alcohol hydroxyl (R─OH), and carbonyl (─CO─). The presence of these functional groups enhances the ability of lignin to participate in diverse chemical reactions [22]. Apart from the multitude of functional groups associated with the structure of lignin units, the chemical activity of lignin is also influenced by the complexity of the side chain structure [23]. The complex arrangement of side chain groups results in a structural formula for lignin that can only be represented by structural models at this stage. However, owing to the variability and instability of the lignin structure, the lignin structure model is the only possible structure that can describe lignin macromolecules [24]. The structure of lignin and the functional model are shown in Figure 2.

### 2.2 Physicochemical Properties of Lignin

**FIGURE 3:**
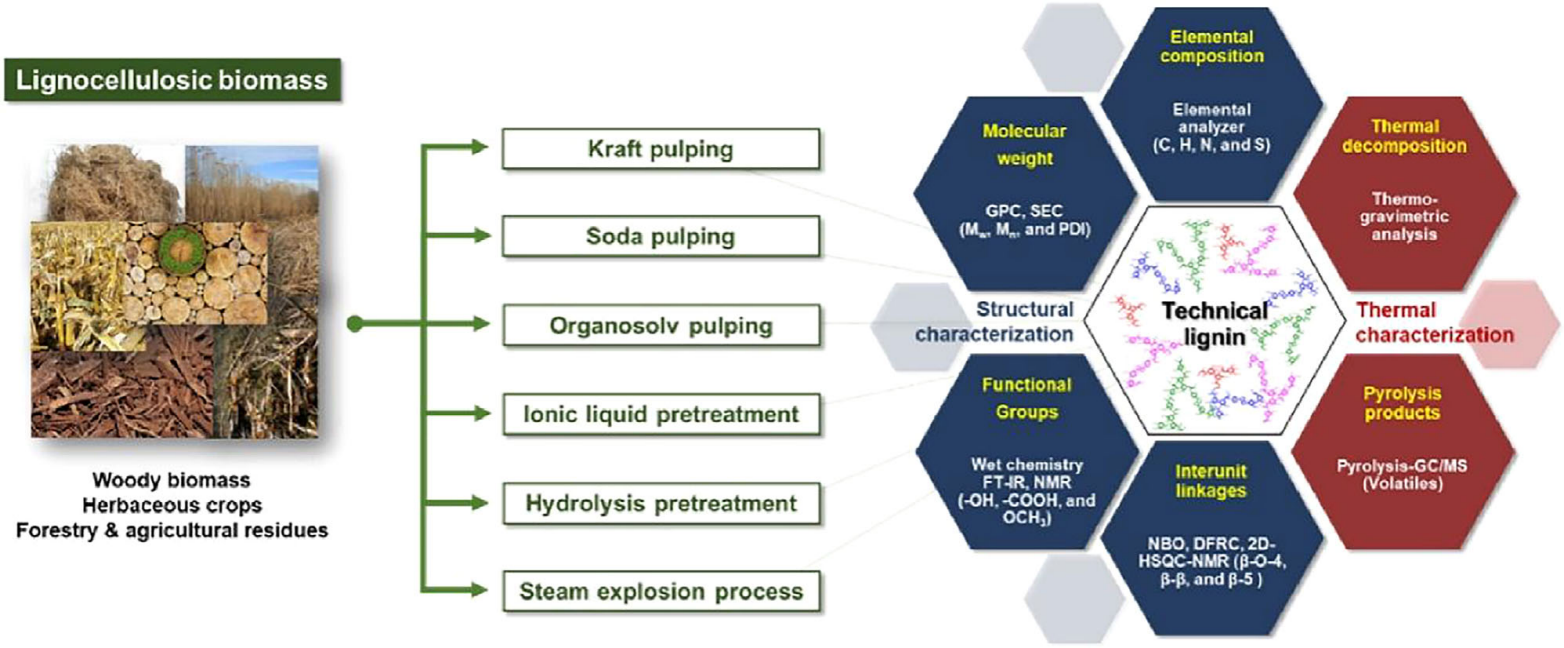
Several kinds of lignin extraction technology and structure [32]. Reproduced with permission of Ref. Copyright 2019, Elsevier.

The structure of lignin is influenced by the plant growth environment and extraction and separation methods, which result in a complex diversity of physicochemical properties of lignin [26]. Figure 3 shows the extraction method for lignin from pulped wood fibers.

Lignin is an almost transparent substance found in plants. However, during the extraction, separation, and preparation of lignin, the structure of lignin is affected by the conditions, resulting in its color deepening and becoming black [27]. Owing to the electron conjugation system formed by the unsaturated double bonds and benzene rings, the absorption spectrum of lignin was redshifted. In addition, the color of lignin darkened in the presence of auxiliary groups such as ─OH, ─NR, ─OR, and ─COOH [28]. Ph─OH, which is present in the monomer structure of lignin, is easily oxidized to quinones in air, which also darkens the color of lignin [29].

The macromolecular structure of lignin comprises numerous active groups, including conjugated double bonds, phenolic hydroxyl, carboxyl, methoxy, alcoholic hydroxyl, and aromatic groups, which enable a diverse range of chemical reactions, such as alcoholysis, oxidation, hydrolysis, acidosis, and reduction [30]. The physical and chemical properties of lignin include its optical, popper, solubility, and thermochemical properties [31]. The chemical characteristics of lignin can be exploited to investigate its structure or utilize lignin for the production of high‐value chemical products.

### 2.3 Application of Lignin

**FIGURE 4:**
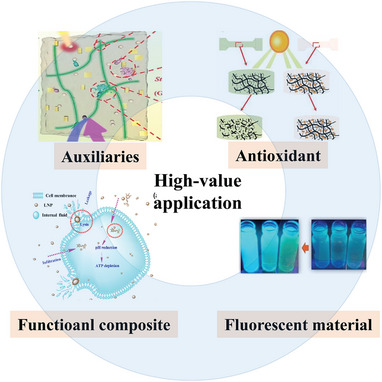
High‐value applications of lignin [36]. Reproduced with permission of Ref. Copyright 2022, Elsevier.

Lignin is a minor product of the pulp and paper industries. More than 95% of lignin is combustible and produces heat. Although combustion can bring value, it causes major waste and environmental pollution [33]. As a result, there is increasing interest in the research and application of lignin aimed at achieving its high‐value utilization [34]. It has immense potential for high‐value applications as antioxidant, adsorbent, additive, drug carrier, nanoparticle, CQDs, and advanced composite polymer material [35]. because it has antioxidant, auxiliary, functional, and fluorescent properties (Figure 4).

#### 2.3.1 Antioxidant Properties

**FIGURE 5:**
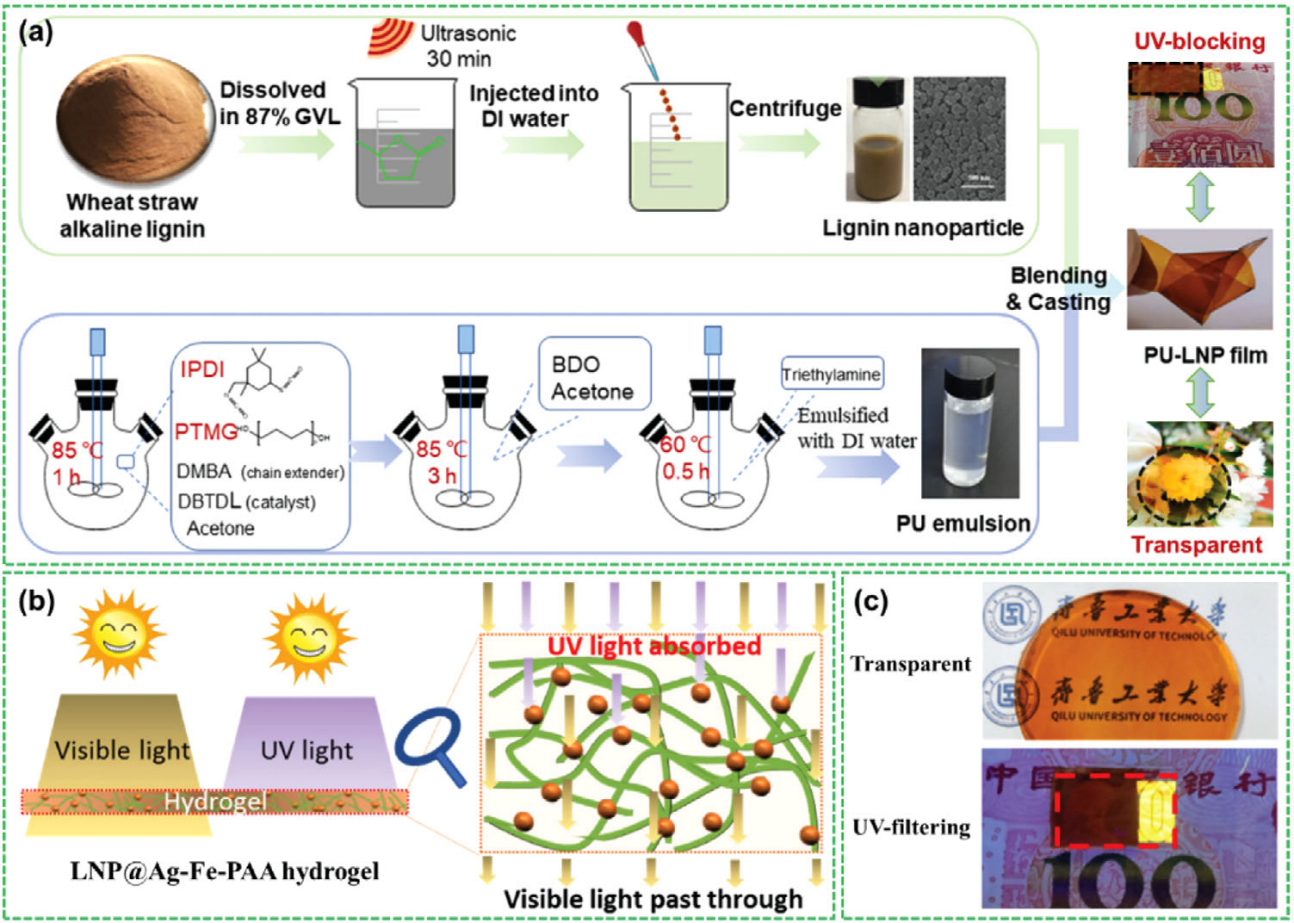
(a) Schematic diagram of synthetic LNP composite membrane [44]; Copyright 2021, Elsevier. (b) LNP@Ag‐Fe‐PAA hydrogel anti‐ultraviolet principle; Reproduced with permission of Ref. Copyright 2022, Elsevier. (c) Picture shows the high transparency of LNP@Ag‐Fe‐PAA hydrogel (top) and covering on the surface of the RMB 100, ultraviolet sensitive fluorescence area water gel UV filtering effect (below) [45]. Reproduced with permission of Ref. Copyright 2022, Elsevier.

Lignin is primarily used as an antioxidant additive in the preparation of packaging bags for food and other products. Bio‐based packaging materials containing lignin have the advantages of being green, safe, harmless, and inexpensive [37]. Lignin has been added to polylactic acid, latex, or starch‐based coatings to improve the antioxidant activity of packaging materials [38]. Jessica et al. [39]. added lignin in polylactic acid and squeeze the film. The experimental results showed that the compounds added to the lignin samples had good oxidation resistance. Crouvisier‐Urion et al. [40]. reported that the incorporation of lignin into a film can scavenge free radicals. Moreover, surface activity was identified as the dominant mechanism responsible for this scavenging effect. Qian et al. [41]. doped lignin nanoparticles (LNP) with skin creams for UV protection and found that LNP‐doped skin creams showed better UV absorption and skin protection than pure lignin‐doped skin creams. When the acetylated lignin formed homogeneous colloidal spheres, the sun‐protection factor of the sunscreen decreased sharply. Tian et al. [42]. synthesized two distinct types of LNP using two different extraction solvents: a deep eutectic solvent and an ethanol‐organic solvent. These NPs were subsequently incorporated into polyvinyl alcohol (PVA) to create a composite film known as an LNP/PVA composite film. Notably, this film exhibited excellent UV shielding capabilities and antioxidant effects. The composite film exhibited good UV shielding and antioxidant effects, and the UV absorption at 400 nm was as high as 80%, with good antioxidant ability. Figure 5 shows the preparation of the LNP composite material, its antioxidation principle, and the application of anti‐counterfeiting (Figure 5) [43].

#### 2.3.2 Auxiliaries

**FIGURE 6:**
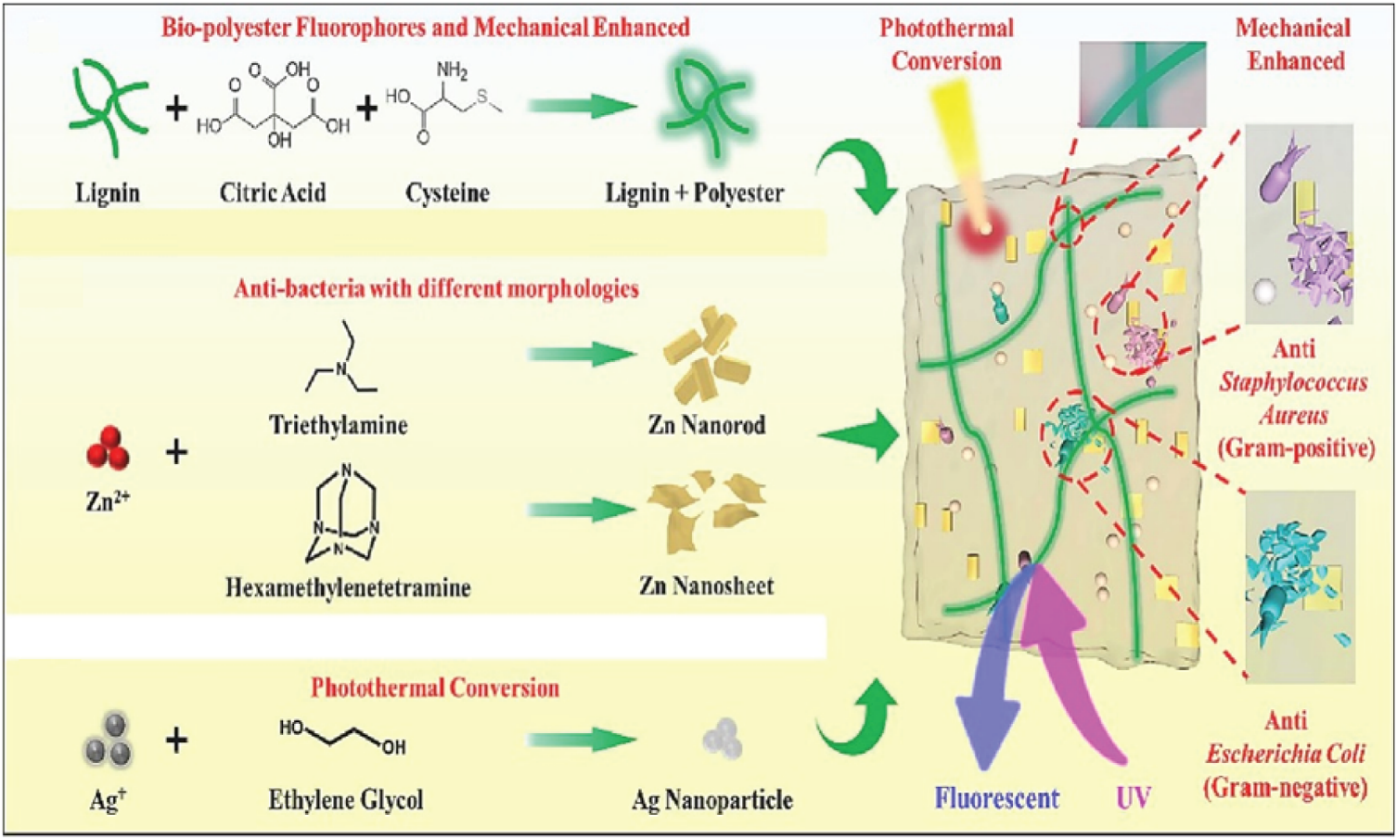
Schematic diagram of multifunctional PLA composites [36]. Reproduced with permission of Ref. Copyright 2021, Elsevier.

Nanoparticles prepared from treated lignin have properties [46] such as oxidation resistance, UV resistance, degradability [47], and particle dispersion, considerably better compared to those obtained from untreated lignin. Hence, numerous investigations have employed LNP as additives to enhance the characteristics of composite materials. Wang et al. [36]. introduced an innovative approach for constructing lignin hybrid modifiers and polylactic acid (PLA) matrices, focusing on enhancing the mechanical properties of PLA rather than on the conventional tradeoff between mechanical strength and functionality. In addition, the fluorescent component is anchored to the lignin surface. Fluorescent molecular moieties can serve as interfacial compatibilizers for complex shape‐tailored blends to promote functional flexibility. Consequently, the PLA composites exhibited remarkable improvements in mechanical strength, fluorescence incorporation, and photothermal conversion efficiency. This combination of enhanced structural integrity, geometric stability, and multifunctional adaptability distinguishes these composites from those used in previous studies. The findings indicated that PLA composites comprising 5% modified lignin, 10% ZnO, and 5% silver exhibited favorable mechanical, fluorescence, and photothermal properties. The multifunctional lignin‐based PLA film was obtained in the present work (Figure 6). Nypel et al. [48]. proposed a new method for assembling lignin into colloids and studied the ability of lignin particles to stabilize cetane Pickering emulsions in aqueous environments. Compared to traditional inorganic particles, lignin nanoparticles have a higher emulsion stabilization efficiency.

#### 2.3.3 Functional Composite

Lignin is widely used as encapsulation and carrier material [49]. Researchers have conducted in‐depth studies on its sustained‐release characteristics, carrier encapsulation efficiency [50] and potential biomedical applications [51].

**FIGURE 7:**
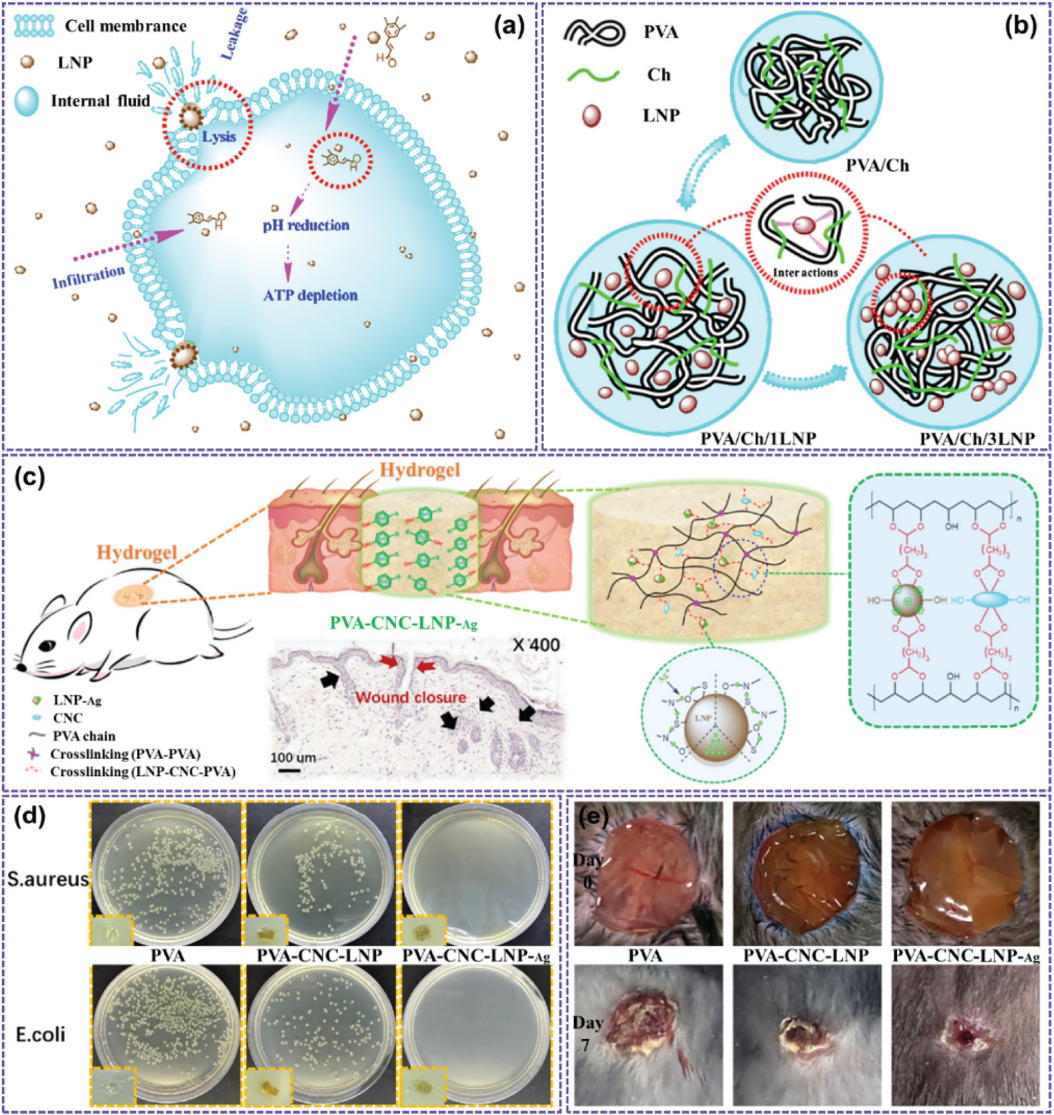
(a) LNP antibacterial behavior mechanism [54]; Copyright 2018, American Chemical Society. (b) Schematic diagram of the interaction between PVA/Ch molecules and LNP [55]; Reproduced with permission of Ref. Copyright 2018, Elsevier. (c) Antibacterial mechanism of LNP composites; (d) Antibacterial effect of polyvinyl alcohol complex hydrogel against gram‐positive *Staphylococcus aureus* and gram‐negative *Escherichia coli* on AGAR plate; (e) Comparison of bactericidal effect of several composite materials [56]. Reproduced with permission of Ref. Copyright 2021, Elsevier.

Yiamsawas et al. [52] synthesized modified lignin by esterifying sulfate‐acidified lignin with methacrylate anhydride. Subsequently, lignin nanocarriers in various forms, such as solid nanoparticles, core–shell structures, and porous nanoparticles, were prepared by microemulsion polymerization and solvent evaporation. The release properties of lignin nanocarriers were studied using a UV‐active drug model. These results showed that the lignin nanocarriers had good slow‐release properties. Versatile and environmentally friendly biodegradable nanocellulars derived from lignin have shown great potential in a range of fields, including agriculture, innovative drug delivery systems, and efficient carbon materials for water purification [53]. Figure 7 shows the slow‐release mechanism of LNPs and the effect of sterilization of the composite materials [43].

#### 2.3.4 Lignin Fluorescence Properties

Natural polymeric fluorescent materials have been used for cell imaging, fluorescence tracers, and sensors because they are renewable, inexpensive, and biocompatible [57]. Plant cell walls exhibit natural photoluminescent behavior, and lignin is considered the main source of fluorescence.

**FIGURE 8:**
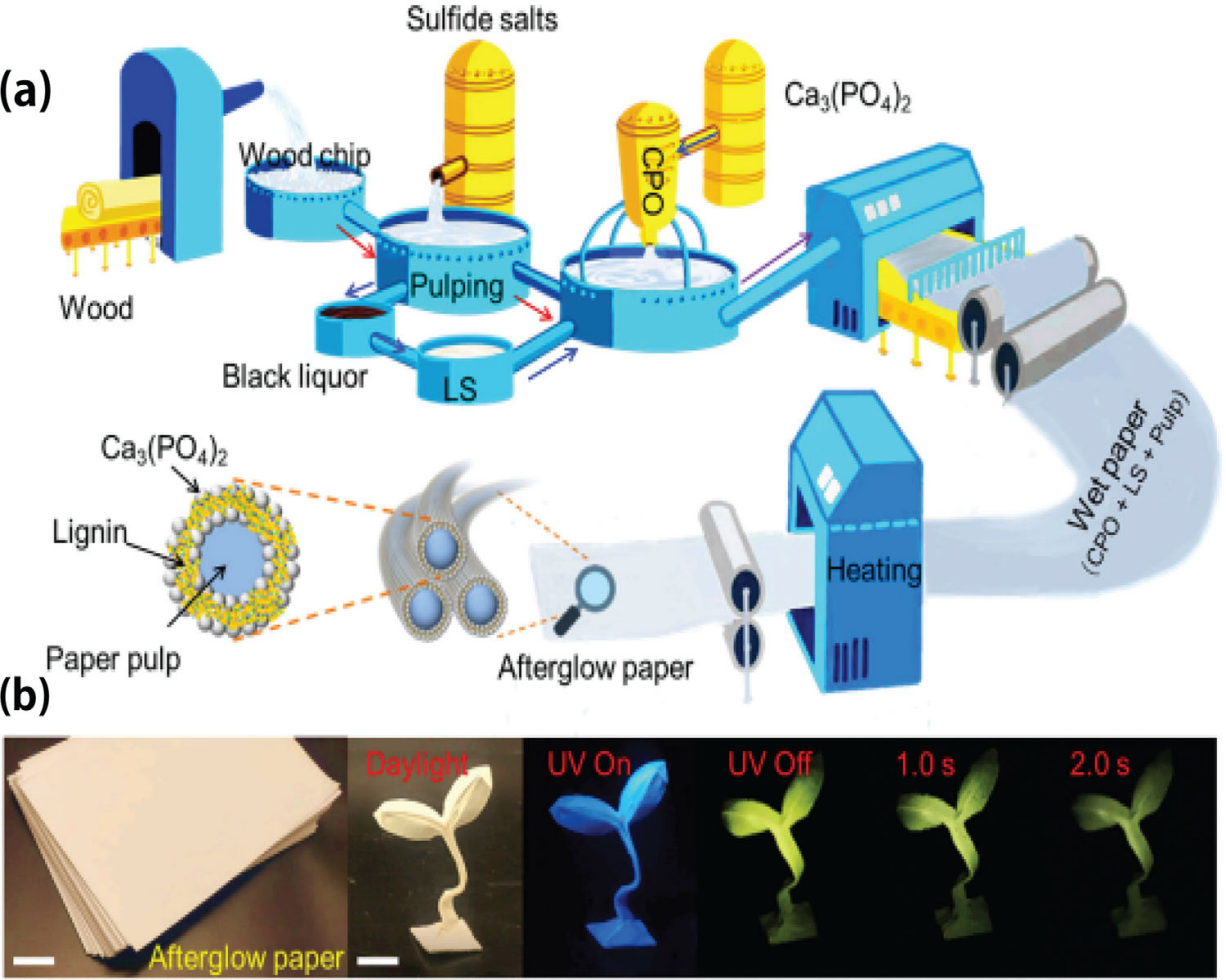
(a) LS@Ca_3_(PO_4_)_2_ Industrial production of aftergloss paper flow chart. (b) Afterglow paper fluorescence effect [58]. Reproduced with permission of Ref. Copyright 2022, Elsevier.

Therefore, considerable attention has been paid to the study of lignin‐based fluorescence properties. Yang et al. [58] found that the prepared afterglow paper had different colors under different wavelengths of light, which is conducive to the application of anti‐counterfeiting performance. Xiong et al. [59] used amidation to graft the fluorescent pyrene molecule onto a lignin group and discussed the potential applications of lignin‐derived fluorescent materials in the field of nanofluorescent sensors. In this synthesis strategy, lignin is used only as a biocompatible polymer carrier material, instead of as the main fluorescent substance. Xue et al. [60] inspired by the fluorescence of wood cell walls, studied the fluorescence behavior of a mixed solvent system comprised of alkali lignin and lignin sulfonate and confirmed the aggregation‐induced emission phenomenon of lignin for the first time. Since then, many researchers have been inspired to prepare reflective materials with fluorescent properties, greatly advancing their research. The industrial process of lignin preparation from afterglow paper and the application of its fluorescence characteristics are shown in Figure 8. The chemical structure of lignin contains many conjugated structural units that emit blue‐green fluorescence under ultraviolet excitation [61].

Several studies have been conducted on the fluorescence properties of lignin [62]. However, the most popular research has been the study of LCQDs, in which the application of LCQDs is more inclined toward anti‐counterfeiting [63]. Therefore, this study focuses on the preparation method and current applications in the direction of anti‐counterfeiting after briefly introducing the applications of lignin.

## 3 Synthesis of CQDs

CQDs are a new class of carbon‐based fluorescent materials that have attracted considerable attention worldwide [64]. Recently, significant progress has been made in the development and utilization of CQDs owing to their excellent performance [65]. A series of new methods and various green, cheap, and abundant raw materials have been widely used for the synthesis of CQDs [66]. The characteristics of the CQDs are primarily affected by their raw materials and preparation methods. Products with different properties can be prepared using various raw materials and methods. Currently, the top‐down and bottom‐up methods are widely used to prepare CQDs. The top‐down synthesis method involves cleaving or destroying a substance containing a carbon‐rich structure by physical or chemical means, and then purifying the surface to give it the ability to produce fluorescence [67].

Arc discharge, laser ablation, and other electrochemical technologies have been widely applied in the preparation of CQDs [68]. Bottom‐up synthesis methods, such as hydrothermal, ultrasonic, microwave, thermal, and decomposition methods, mainly use small organic or aromatic molecules as precursors to obtain fluorescent CQDs through pyrolysis or carbonization [69].

**FIGURE 9:**
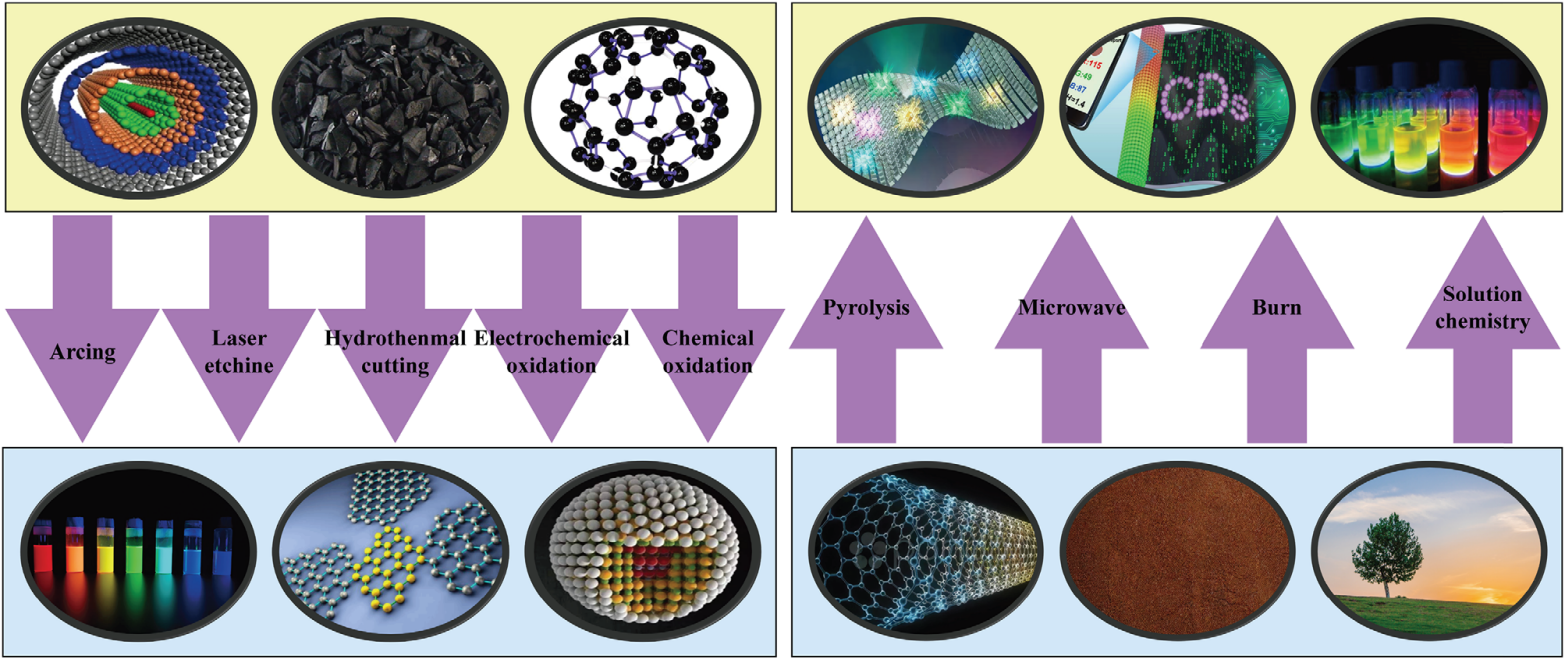
Top‐down and bottom‐up methods for preparing CQDs.

Lignin, a natural polymer, has significant advantages in the preparation of CQDs. First, lignin, a by‐product of the pulp and paper industry, is widely sourced and renewable, which keeps its cost low and enhances its economic feasibility [70]. Second, the chemical structure of lignin is rich in aromatic rings and carbon, providing an ideal precursor for the production of high‐quality CQDs. In addition, the environmental friendliness of lignin effectively reduces the dependence on fossil fuels and environmental pollution [71]. Additionally, the chemical modification of lignin is relatively straightforward, and the size, shape, and dispersion of CQDs can be controlled by different chemical and physical methods, such as hydrothermal synthesis and microwave‐assisted synthesis, thereby regulating the optical properties of lignin‐based quantum dots [72]. Lignin has been extensively utilized in the synthesis of CQDs in many fields such as bioimaging, ion sensing, luminescent materials, and photocatalysis [73]. Schematics of the top‐down and bottom‐up preparation of CQDs are shown in Figure 9.

So far, a variety of methods have been developed to prepare LCQDs, which can be convenient, fast, customized and high yield, large‐scale synthesis of high‐quality LCQDs [74]. Typical methods for synthesizing LCQDs from lignin are hydrothermal and microwave methods.

### 3.1 The Hydrothermal Synthesis Technique

The hydrothermal method, also known as the hydrothermal carbonization (HTC) process, is a widely utilized bottom‐up approach for the synthesis of CQDs. This approach offers several advantages, including simple and accessible preparation of precursors, simple and controllable reaction solvents, and low contamination. HTC can be used to synthesize LCQDs with uniform size and good dispersion at low temperatures and pressures. LCQDs of different sizes and optical properties were obtained by changing the synthesis conditions. HTC is widely recognized as a straightforward, direct, and efficient strategy for the synthesis of CQDs.

Hao et al. [75] prepared CQDs from kraft lignin using HTC and observed that they exhibited significant fluorescence properties. Chen et al. [76] (2016) obtained blue incandescent CQDs for the first time using lignin as the carbon source and hydrothermal treatment with H_2_O_2_. Although the yield of the generated CQDs was low, the synthesized CQDs had good photostability, providing a new concept, development, and direction for the preparation of CQDs.

Ding et al. [77] developed a two‐stage method for preparing CQDs using alkaline lignin as a carbon precursor system. The prepared CQDs can stimulate a specific wavelength of bright green light under ultraviolet irradiation and can stimulate wavelength dependence, up‐conversion, and other behaviors, providing new prospects for the application of fluorescence probes in the infrared field. Gao et al. [12] used alkali lignin and a mixed acid to synthesize CQDs using a one‐step hydrothermal method, and the experimental results confirmed that the yield significantly increased to 20%.

**FIGURE 10:**
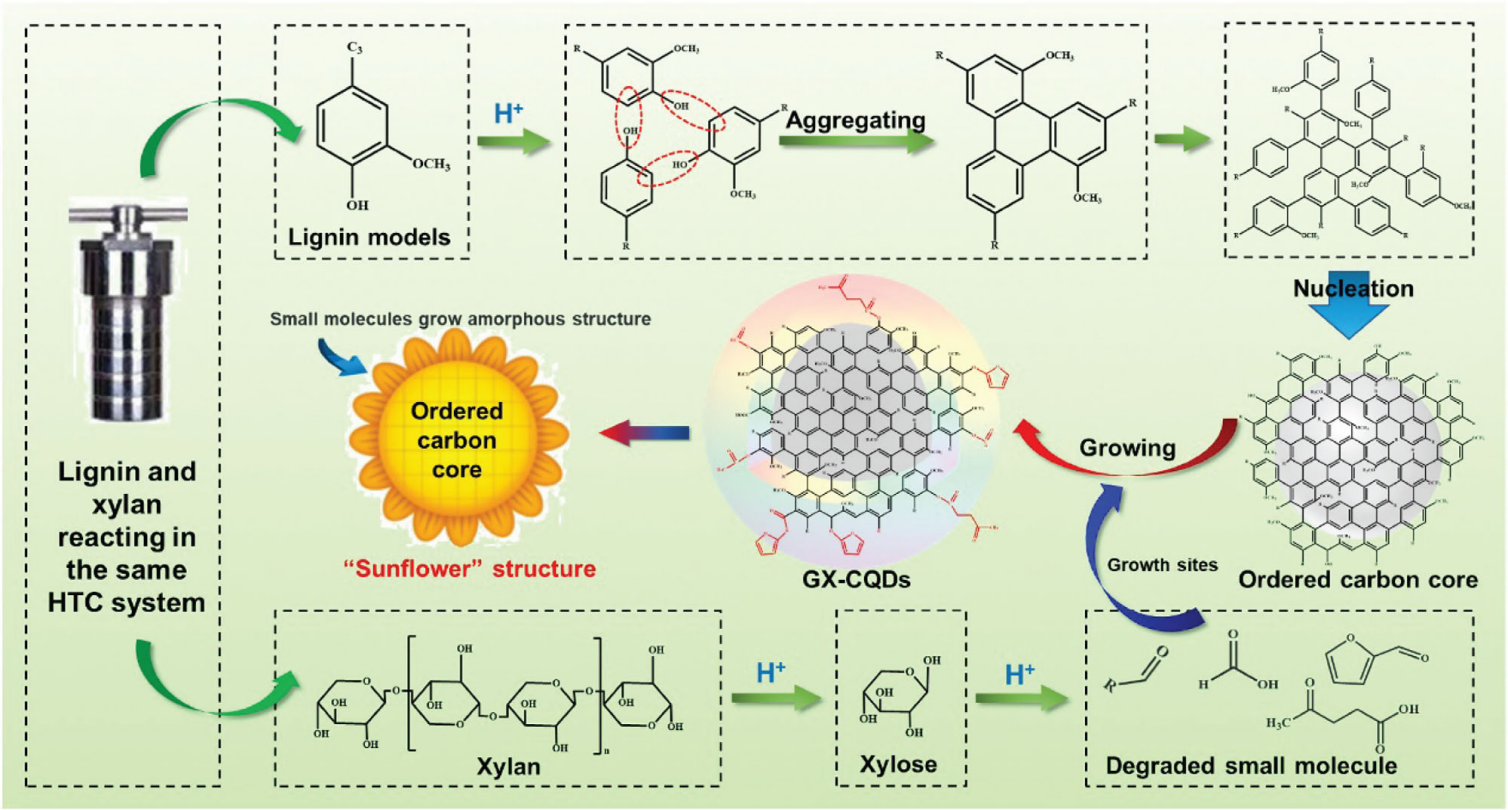
CQDs prepared hydrothermal method and performance characterization [78]. Reproduced with permission of Ref. Copyright 2022, Elsevier.

Zhao et al. [78] prepared LCQDs with excellent in vivo imaging and fluorescence characteristics using a hydrothermal method. LCQDs of different sizes can be synthesized using simple, environment‐friendly, and effective methods. To obtain fluorescent LCQDs, concentrated oxidizing acids (such as sulfuric, nitric, and hydrochloric acids) are used to oxidize and break the carbon source. By modifying the surface functional groups, the obtained CQDs exhibited fluorescence properties. The operation process has a certain risk, and the formed acidic and alkaline waste liquids pollute the environment. Figure 10 shows the preparation of carbon using the hydrothermal method and the characterization of their fluorescence properties.

### 3.2 Microwave Synthesis Method

Microwave synthesis has the advantages of being simple, fast, green, pollution‐free, and low‐cost [79]. Currently, the microwave method is the most suitable method for the large‐scale synthesis of CQDs.

Zhu et al. [80] prepared CQDs using a microwave method. First, sugar was dissolved in ethylene glycol, and microwave heating was used to promote the reaction. As the reaction progressed, the color of the solution changed from transparent to yellow, and finally to dark brown. This change indicates that a carbonization reaction occurred, resulting in the formation of CQDs. Although CQDs were obtained by the separation of different wavelengths, the yield was very low. Wang et al. [81] used catechol, hydroquinone, and resorcinol as precursors to synthesize fluorescent CQDs with size distribution between 0.5 and 6 nm by microwave heating, which increased the yield by 42.8%. Ma et al. [82] synthesized LCQDs by microwave heating and combined them with carbon nanosheets to prepare a composite material with high electrical conductivity. Si et al. [62] proposed a microwave‐assisted rapid synthesis of LNP and LCQDs. In their study, lignocellulosic straw, as a carbon source, was subjected to a 400 W microwave reaction for 10 min under the action of an ethanol‐water system and an acid catalyst. The LNP and LCQDs were obtained via high‐speed centrifugation. Subsequently, with the assistance of microwaves, small molecules such as monosaccharides were synthesized into LCQDs by dehydration, polymerization, aromatization, and carbonization. Rai et al. [83] confirmed that lignin‐derived reduced‐fluorescence fluorescence carbon dots exhibited an impressive quantum yield of 47.4%, demonstrating their potential utilization as drug carriers.

**FIGURE 11:**
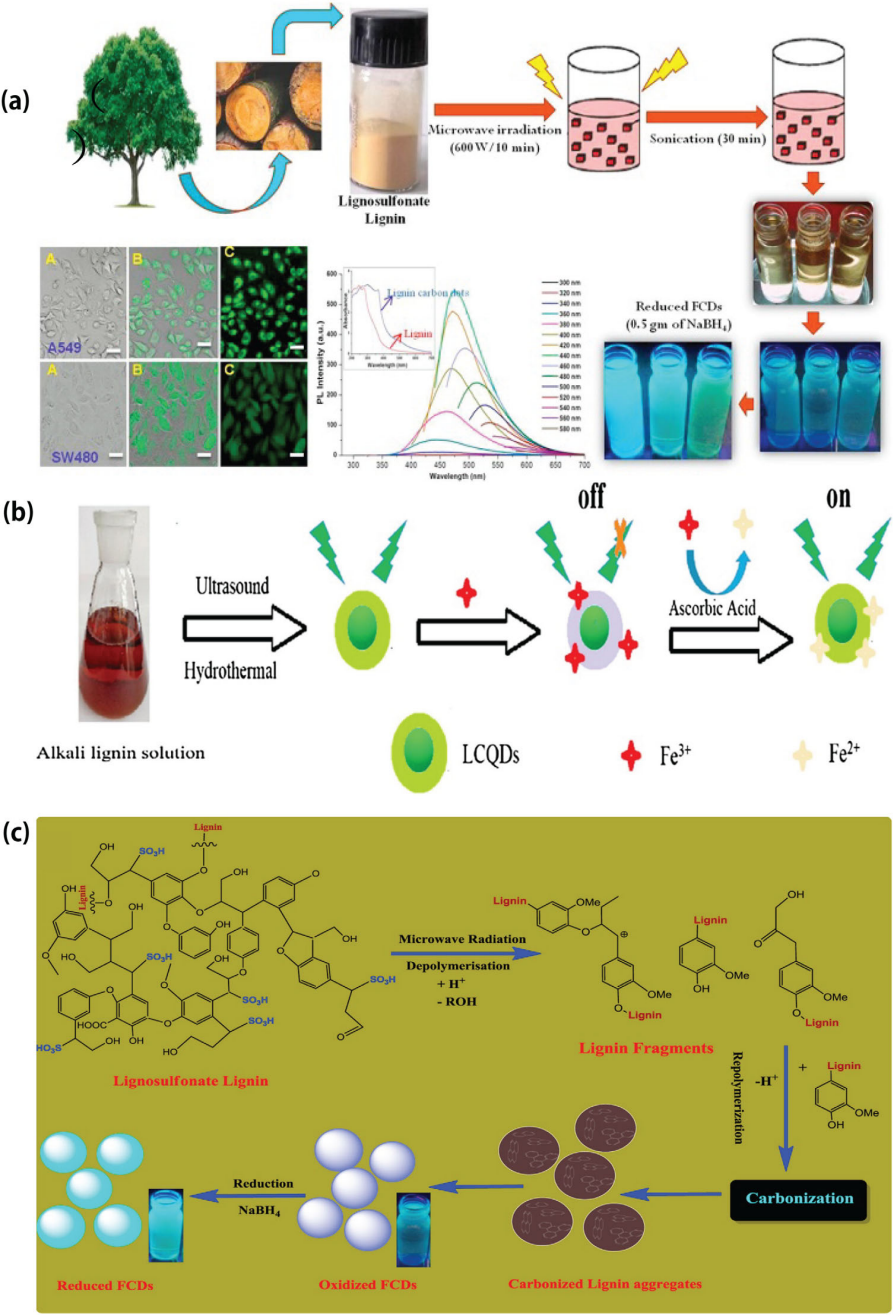
(a) LCQDs were synthesized by microwave [12, 83]. (b) LCQDs with ionic response characteristics were prepared by microwave method [80]. Copyright 2018, Elsevier. (c) LCQDs schematic diagram of fluorescence characteristics prepared by microwave method [12]. Reproduced with permission of Ref. Copyright 2017, Elsevier.

In summary, the microwave method is an efficient method for synthesizing CQDs with the advantages of simple operation, low cost, high fluorescence quantum yield, easy access to raw materials, and no need for complex equipment. However, the disadvantage is that the particle size distribution is not uniform, and further separation and purification are required. Figure 11 shows a flowchart of the LCQDs prepared using the microwave method (Figure 11).

### 3.3 Ultrasound Synthesis Method

The ultrasonic synthesis method uses the thermal effect generated by ultra‐high‐frequency vibrations to promote the reaction, which has significant advantages in terms of environmental protection, economy, strong penetration, and consistent results [84, 85]. Carbon dots (CDs) obtained by the traditional carbonization method often have large particle sizes and uneven surface morphologies [86]. Therefore, the combination of ultrasonic synthesis with other methods can confer excellent performance.

**FIGURE 12:**
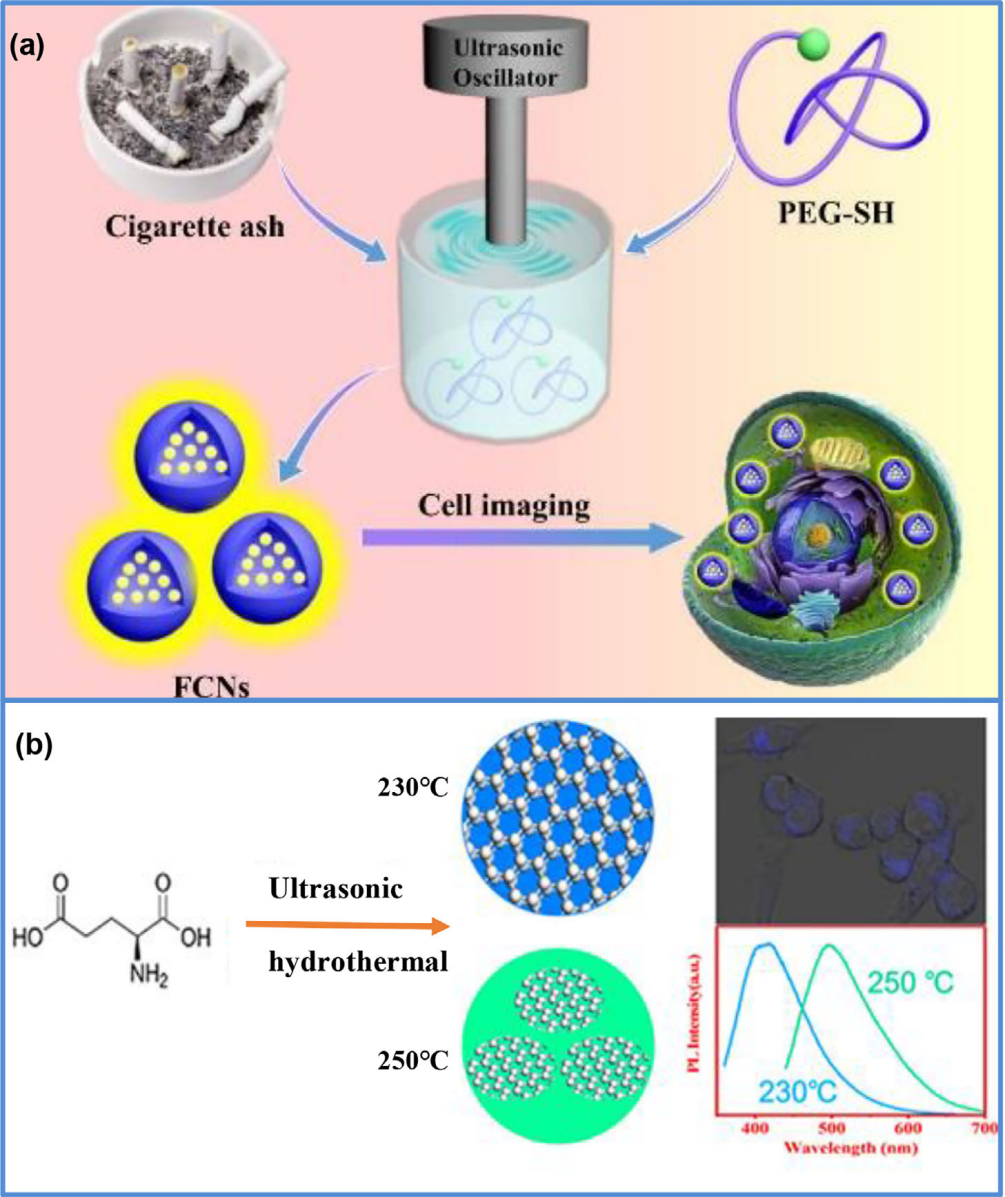
(a) CQDs were synthesized by ultrasonic method [87, 88]. Reproduced with permission of Ref. Copyright 2018, Elsevier. (b) LCQDs were prepared by microwave assisted hydrothermal method [85]. Reproduced with permission of Ref. Copyright 2021, American Chemical Society.

Huang et al. [87] were the first to successfully synthesize CDs using smoke as a precursor. Under ultraviolet irradiation at 365 nm, the CDs exhibited bright green fluorescence. Qi et al. [88] synthesized two types of doped CQDs with different morphologies and structures using ultrasonic‐assisted synthesis. The results show that The synthesized CQDs exhibited excellent optical properties, high yield, strong photobleaching resistance, and excellent photostability. The preparation of CQDs via the ultrasonic method using different raw materials is illustrated in Figure 12.

Notably, there have been few reports on the synthesis of CDs using ultrasonic methods. Compared to direct or microwave heating, the local thermal effect generated by ultrasound may lead to uneven heating and a lower overall reaction efficiency. However, there are still some challenges and much room for improvement in ultrasonically synthesized CDs. Despite the simplicity and cost‐effectiveness of the ultrasonic equipment, the reported yields for the preparation of CQDs are still relatively low, and the yields of synchronized optical quantum reactions are limited and need to be broadly addressed.

### 3.4 The Chemical Oxidation Technique

Chemical oxidation is a new method for synthesizing CDs, which usually uses a strong oxidizing agent, such as sulfuric acid or nitric acid, to oxidize carbon precursors. CDs prepared by chemical oxidation contain more functional groups owing to the oxidation reaction; therefore, they have broader application prospects [89].

Bao et al. [90] used different concentrations of nitric acid to oxidize carbon fibers and manipulated the reaction temperature and time, the degree of graphitization of carbon nuclei, and the degree of surface oxidation of the prepared CQDs. The experimental results confirmed the successful synthesis of multicolor fluorescent CQDs. Hu et al. [91] put forward the idea of utilizing H_2_O_2_ as an oxidant instead of acid in the synthesis of CDs with remarkable photocatalytic activity.

**FIGURE 13:**
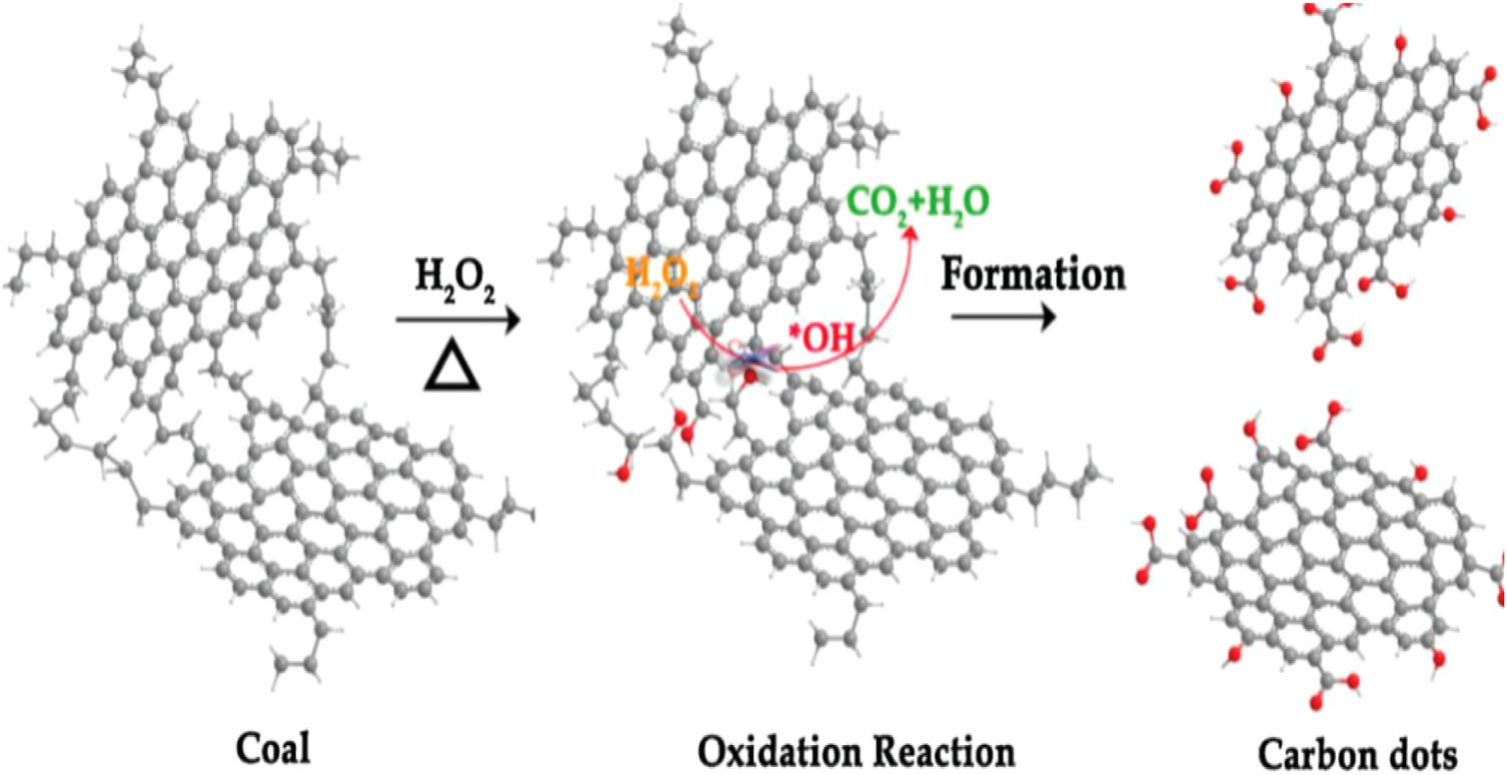
CQDs were synthesized by chemical oxidation [91]. Reproduced with permission of Ref. Copyright 2018, Elsevier.

Although CDs prepared by chemical oxidation exhibit excellent photoluminescent properties, their potential chemical toxicity should not be neglected. The quantum yield of CQDs prepared by the oxidation method is low, and surface passivation is required to improve the fluorescence quantum yield, which often requires chemical reagents and is time‐consuming [92]. However, chemical oxidation is an efficient and straightforward method for the large‐scale preparation of CQDs that eliminates the need for complex synthesis equipment. The diagram shows a flow chart of the CQDs preparation by chemical oxidation (Figure 13).

### 3.5 Laser Etching Method

Laser etching is a technique commonly used for the synthesis of CQDs. LCQDs with uniform size and regular shape can be prepared by laser etching. The etching depth and formation of carbon points can be controlled by adjusting the power, speed, and exposure time of the laser, and the optical properties and electronic structure of the LCQDs can be adjusted. However, this process must be performed under the protection of argon gas, and the synthesized products must be surface‐modified to form fluorescent CQDs [93].

Sun et al. [94] reported the preparation of CQDs by laser etching for the first time. Hu et al. [95] precisely controlled the size of CQDs by changing the laser pulse width and adjusting the contact interaction between the laser beam and the graphite sheet. This adjustment affected the formation of graphite nuclei by modifying the interfacial environment. However, experimental results showed that the synthesized CQDs did not have direct luminescence characteristics and required surface modifications.

**FIGURE 14:**
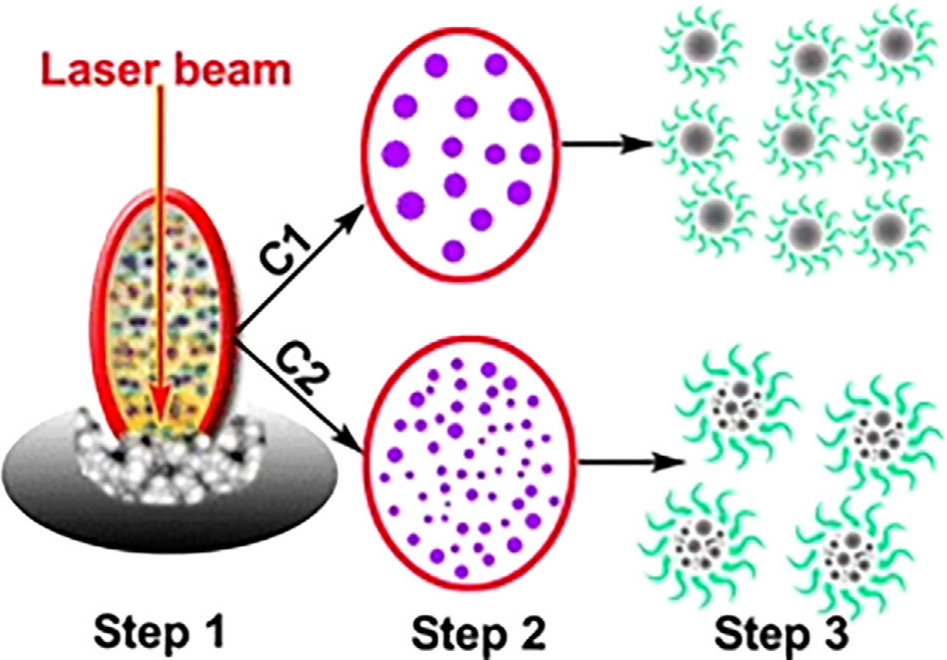
Preparation of CQDs by laser etching [95]. Reproduced with permission of Ref. Copyright 2011, Springer Science.

Nonetheless, a significant advantage of this method is the ability to control and regulate the size of the CQDs by simply adjusting the width of the laser pulse. Figure 14 shows the preparation of CQDs by laser etching (Figure 14).

### 3.6 Electrochemical Oxidation Method

Electrochemical oxidation is frequently employed as a popular method for the top‐down synthesis of CQDs. The principle of electrochemical oxidation is to electronically oxidize water to produce a large number of hydroxyl radicals, superoxide ions, and other active oxidation ions. Generally, carbon materials are used as the anode of the working electrode, and carbon nanomaterials, such as carbon nanotubes [96], graphite [97], coke [98], and graphite paper [99] are stripped from the electrode after working at a certain voltage for a period of time. Electrochemical oxidation can be used to control the size and dispersion of LCQDs by adjusting the parameters of the process to prepare high‐purity LCQDs. Then, CQDs with fluorescence emission characteristics were obtained by surface passivation treatment.

**FIGURE 15:**
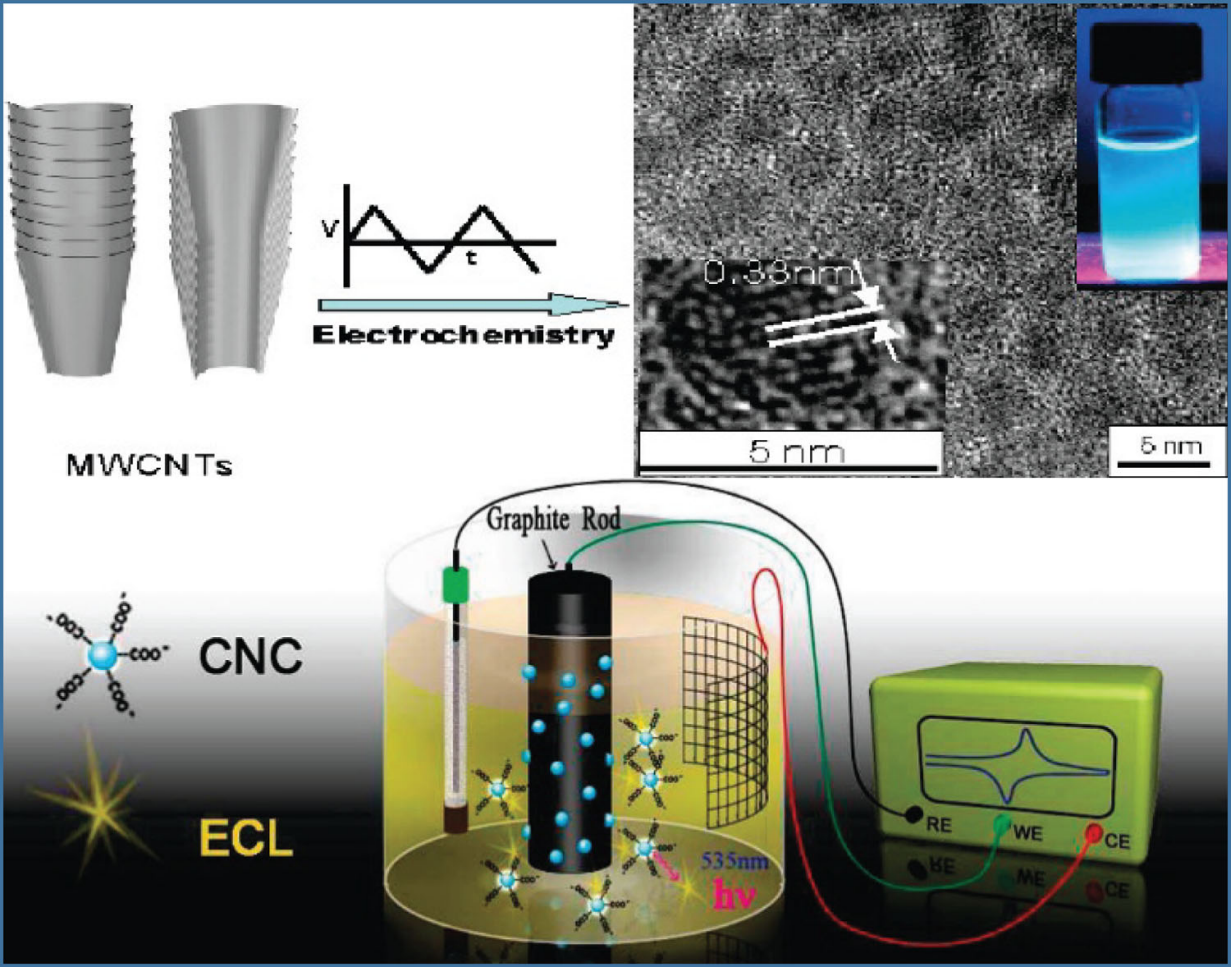
Preparation of CQDs by electrochemical oxidation [100, 101]. Reproduced with permission of Ref. Copyright 2007, American Chemical society. And Copyright 2009, American Chemical society.

Zhou et al. [100]. used multiwalled carbon nanotubes as the working electrode and a platinum wire as the reverse electrode. By electrolyzing a solution of tert‐butyl ammonium perchlorate in acetonitrile, researchers successfully stripped fluorescent CQDs from CNTS with an average size of approximately 2.8 nm and a yield of approximately 6.4%. Zheng et al. [101]. prepared CQDs with similar sizes and high crystallinity via electrochemical oxidation using ethanol as the carbon source under alkaline conditions. The results show that the CQDs have a pH‐sensitive response and can be used as probes for pH detection. The synthesis of CQDs via electrochemical oxidation is an economical, simple, efficient, reproducible, and high‐yielding method. However, this method is limited by certain conditions of electrolyte selection and carbon source. Nevertheless, this is still a favorable method for preparing CQDs. Figure 15 shows the preparation of CQDs by electrochemical oxidation (Figure 15).

## 4 Fluorescence Properties Application

LCQDs have a wide range of applications owing to their excellent photoluminescence (PL) properties [102]. First, the strong fluorescence of LCQDs can be excited in the visible spectrum, which provides them with potential applications in the fields of biological imaging, sensing, and optical labeling. Second, the fluorescence emission wavelength of the CQDs can be modulated by changing the excitation wavelength, pH, and size, thereby providing the possibility for the development of materials with specific luminescence characteristics [103]. The excellent luminous efficiency of the LCQDs can be attributed to their high fluorescence intensity and relatively large quantum yield. Moreover, LCQDs exhibit good water dispersibility and biocompatibility, which are particularly important for biomedical applications [104]. Finally, the fluorescence characteristics of LCQDs are stable, and consistent luminous performance can be maintained even under harsh conditions, such as high temperatures, which ensures their reliability in practical applications [105]. In conclusion, the comprehensive fluorescence characteristics of the LCQDs indicate their broad potential in the field of fluorescence.

**FIGURE 16:**
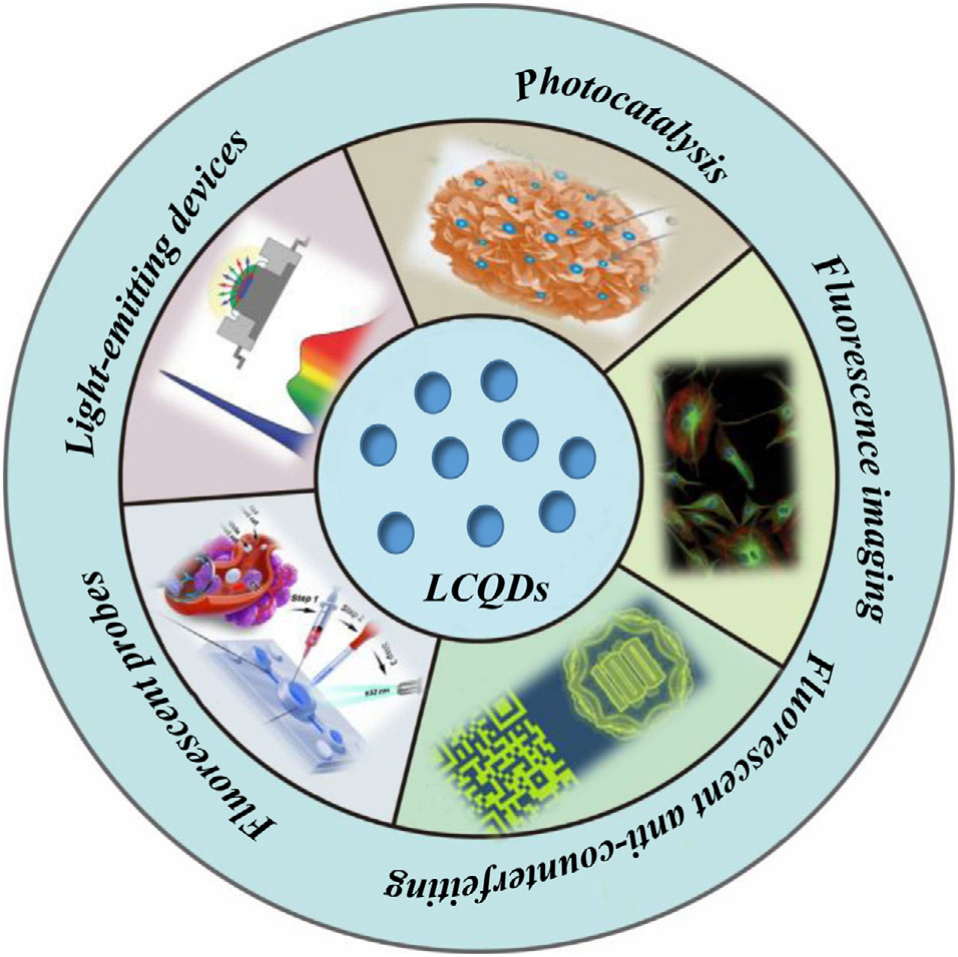
Application of fluorescence characteristics of LCQDs.

CQDs, which exhibit excellent biocompatibility, photochemical stability, photoluminescence (PL) tunability, and easy surface functionalization, provide unprecedented opportunities for fluorescence imaging, disease diagnosis, treatment, and probe detection [103]. The applications of LCQDs in fluorescence imaging, fluorescence probes, fluorescence anti‐counterfeiting, light‐emitting devices, and photocatalysis are shown in Figure 16.

### 4.1 Fluorescence Imaging

CQDs provide a solid foundation for a variety of cell‐imaging applications owing to their small particle size, high water solubility, impressive fluorescence properties, and biocompatibility [106]. Currently, the combination of CQDs and optical imaging technology is an important research direction for biological imaging and biomarkers [107].

**FIGURE 17:**
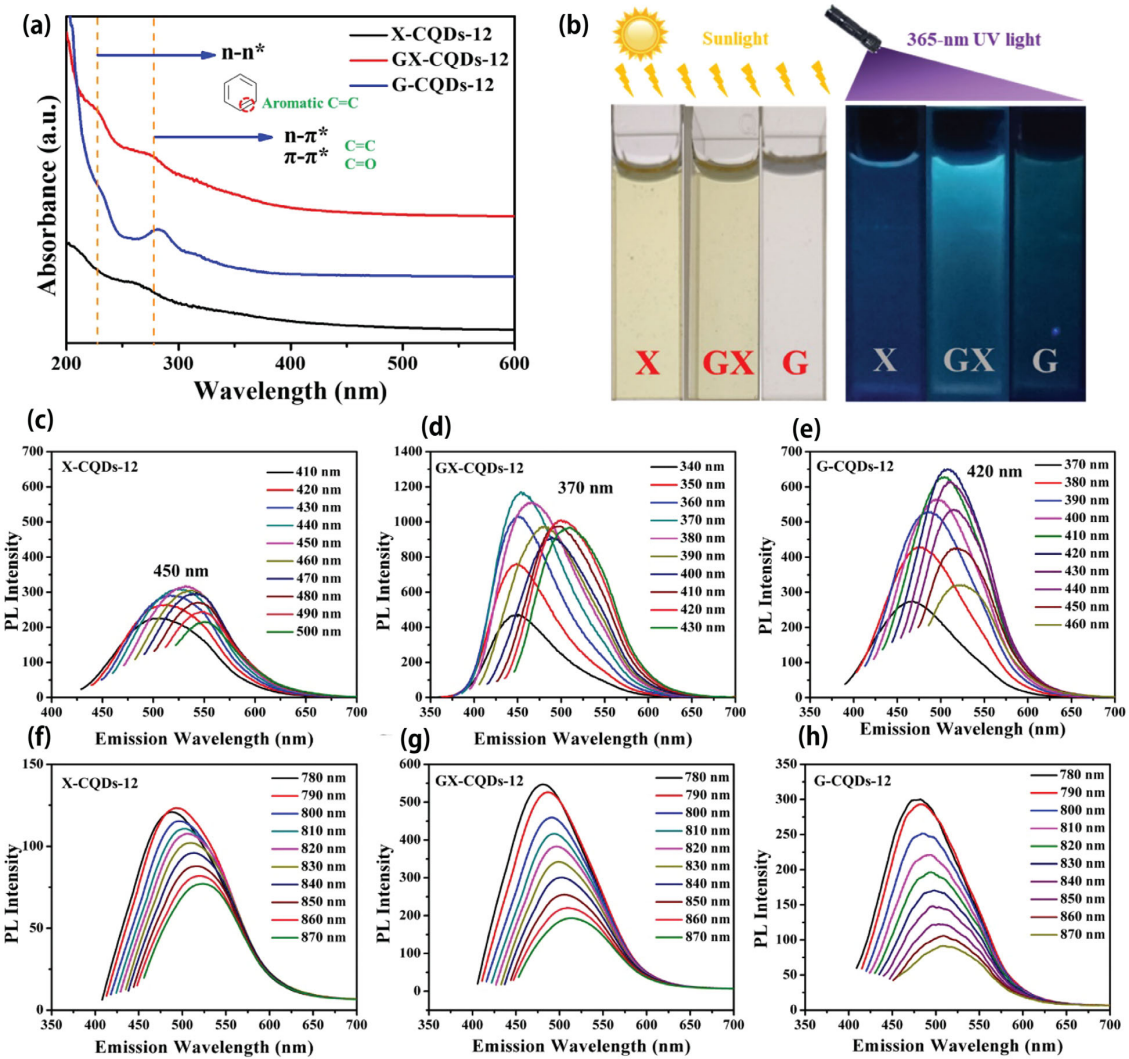
(a) Ultraviolet–visible (UV–vis) spectroscopy analysis of carbon quantum dots derived from lignin. (b) Photographs of G‐CQDs‐12, S‐CQDs‐12, and H‐CQDs‐12 under sunlight and 365 nm UV light. PL spectra of (c) G‐CQDs‐12, (d) S‐CQDs‐12, and (e) H‐CQDs‐12. Up‐PL spectra of (f) G‐CQDs‐12, (g) S‐CQDs‐12, and (h) H‐CQDs‐12 [78]. Reproduced with permission of Ref. Copyright 2022, Elsevier.

Zhao et al. [78]. studied the hydrothermal degradation process of lignocellulose and prepared “sunflower” CQDs by HTC. The results showed that The prepared CQDs exhibited a good in vivo imaging performance. Vaibhavkumar et al. [108]. used a simple green hydrothermal method to synthesize CQDs with bright blue fluorescence emissions from apple juice. The results showed that The synthesized CQDs exhibited good imaging effects and biocompatibility with bacteria and fungi. In addition, the CQDs did not impede cell growth, further supporting their biocompatibility. Subsequently, Christy [109] successfully prepared malic acid‐based CQDs with good biocompatibility using malic acid as the main material. The synthesized CQDs were suitable for super‐resolution fluorescence localization microscopy imaging under different conditions. In 2009, Yang et al. [110]. prepared PEG‐oligomerization‐passivated CQDs for the first time. The experimental results show that the synthesized CQDs have good biocompatibility and are effective fluorescent developers. Zhou et al. [111]. synthesized CQDs using watermelon rind as a raw material via a low‐temperature carbonization process and demonstrated their applicability in imaging human cervical cancer cells. Lin et al. [112]. observed the uptake of CQDs by MCF‐7 human breast cancer cells in the process of co‐culturing with CQDs and observed that the CQDs emitted strong fluorescence signals under a fluorescence microscope. The preparation of CQDs with good fluorescence imaging effect is shown in Figure 17.

### 4.2 Fluorescent Probes

The good biocompatibility and low toxicity of CQDs render them ideal materials for biomedical research. LCQDS for biological detection are required to be less than 10 nanometers in size in order to be able to penetrate various biological barriers in the body [113]. Furthermore, the surfaces of these carbon dots are covered with a layer of polymer groups that can be chemically modified to enhance their stabilities and targeting capabilities. The fluorescence properties of LCQDs depend on the excitation and pH stabilization, which are essential for accurate cell imaging and biological detection [114]. The electronic structure and optical properties of LCQDs can be adjusted by doping with heteroatoms, which optimizes their applications in biosensing and imaging [115].

For example, CQDs can produce different colors under different conditions. Owing to these properties, CQDs can be used as highly sensitive fluorescent probes [116]. Since 2016, CQDs prepared from lignin have become popular in the field of cell labeling owing to their large specific surface area, good water solubility and dispersion, and excellent fluorescence performance. By doping or functionalizing heterogeneous elements such as N, S, and P, different chemical groups can be connected to LCQDs. This enables them to interact with a wide variety of substances. Consequently, the fluorescence intensity experienced a substantial linear burst. Based on this property, LCQDs have the potential to replace traditional toxic semiconductor quantum dots and provide an environmentally friendly method to analyze and detect metal ions, small molecules, and other substances.

**FIGURE 18:**
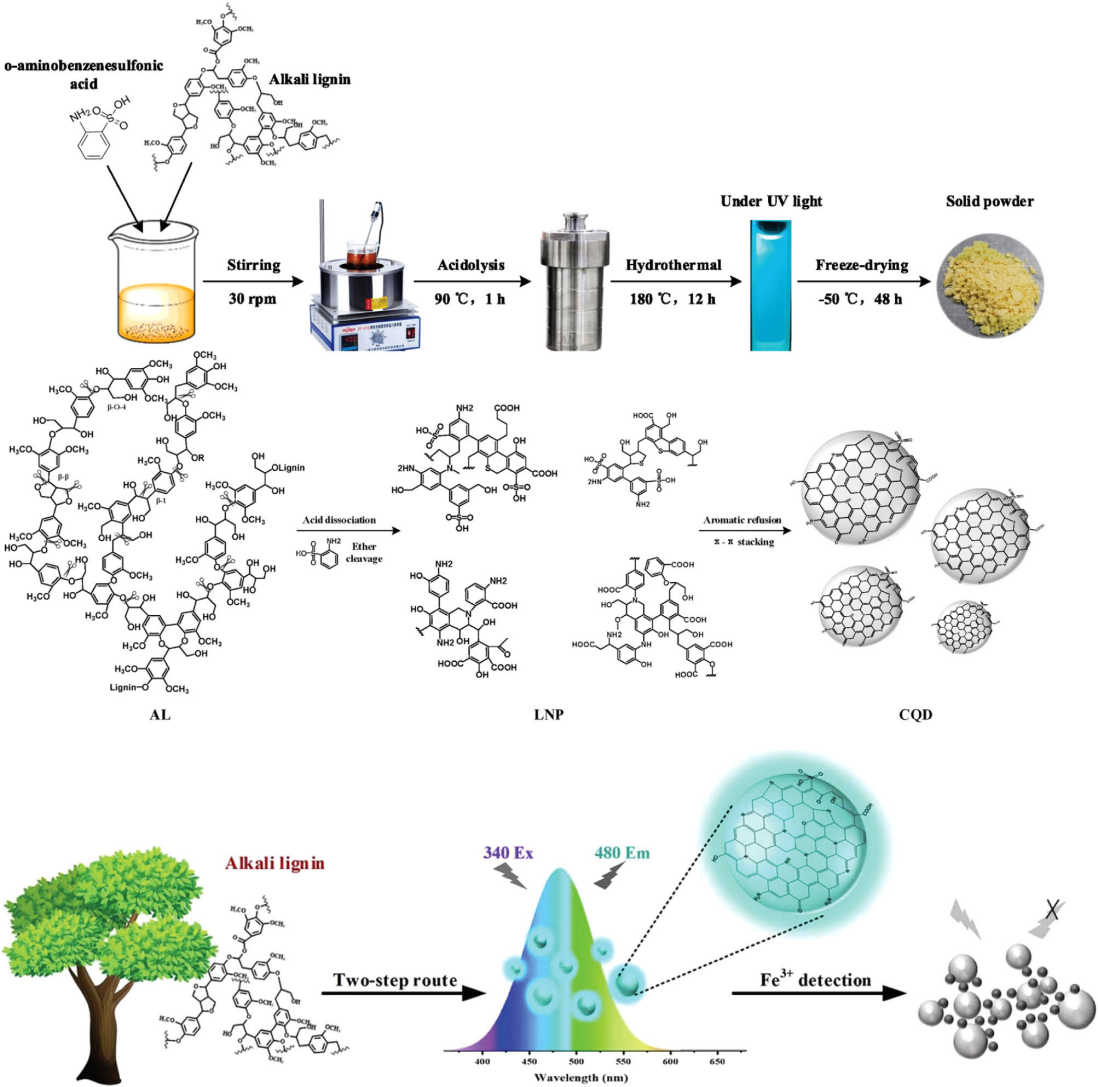
Synthesis and doping diagram of LCQDs [116]. Reproduced with permission of Ref. Copyright 2021, Elsevier.

Shi et al. [105]. synthesized nitrogen‐doped LCQDs using alkali lignin as the carbon source by introducing secondary amine groups into the main chain of lignin. It was found that the LCQDs have a strong linear response to ferrous ions and can be used for the quantitative measurement of ferrous ions. Zhang et al. [7]. successfully prepared LCQDs with green fluorescence via a hydrothermal method using alkali lignin and ethylenediamine as raw materials. The prepared LCQDs exhibited significant and specific fluorescence reactions with Ag ions. In addition, high sensitivity was observed for the other tested ions. The fluorescence efficiency linearly and positively correlated with the silver ion concentration, with a correlation coefficient of 0.992. Compared to existing detection methods for silver ions, these LCQDs have the advantages of a low detection limit and wide detection range, providing a highly sensitive and rapid platform for the detection of silver ions. Wang et al. [117]. prepared N‐doped‐ and S‐doped LCQDs with o‐aminobenzene sulfonic acid, and the results showed that the LCQDs had significant selectivity for the physiological oxidant, H2O2. This provides a new method for small molecule detection and a new way for exploration in this field. A schematic of the preparation of the fluorescent probe with good results is shown in Figure 18.

### 4.3 Light‐Emitting Devices

LCQDs, which are used to make optical devices, typically consist of a carbon core less than 10 nm in size, which is highly carbonized and rich in carbon and hydrogen, as well as heteroatoms such as nitrogen or sulfur [61]. LCQDs doped with heteroatoms have different electronic structures and optical properties and can be customized for specific applications. Zhang et al. [118]. produced polymerized fluorescent carbon nanodots (PCND) by microwave‐assisted pyrolysis and surface fermentation. The PCNDs were then combined with methacrylic acid via copolymerization to form a translucent and vibrant blue 3D fluorescent macrocomposite. Atchudan et al. [119]. synthesized fluorescent nitrogen‐doped carbon quantum dots (FNCDs) using West Indian vinegar as the new carbon source and ammonia as the nitrogen source. The obtained FNCDs exhibited strong fluorescence, good biocompatibility, and a high quantum yield.

Xiang et al. [120]. successfully synthesized CQDs with multicolor luminescence via controlled pyrolysis. Homogeneous dispersion of CQDs in epoxy resin was demonstrated, resulting in a transparent CQD/epoxy composite material. Notably, these composites show great potential as multicolor and white‐light‐emitting devices. This breakthrough lays the foundation for exploring the use of cost‐effective CQDs as alternatives to phosphors in multicolor white‐light‐emitting devices.

**FIGURE 19:**
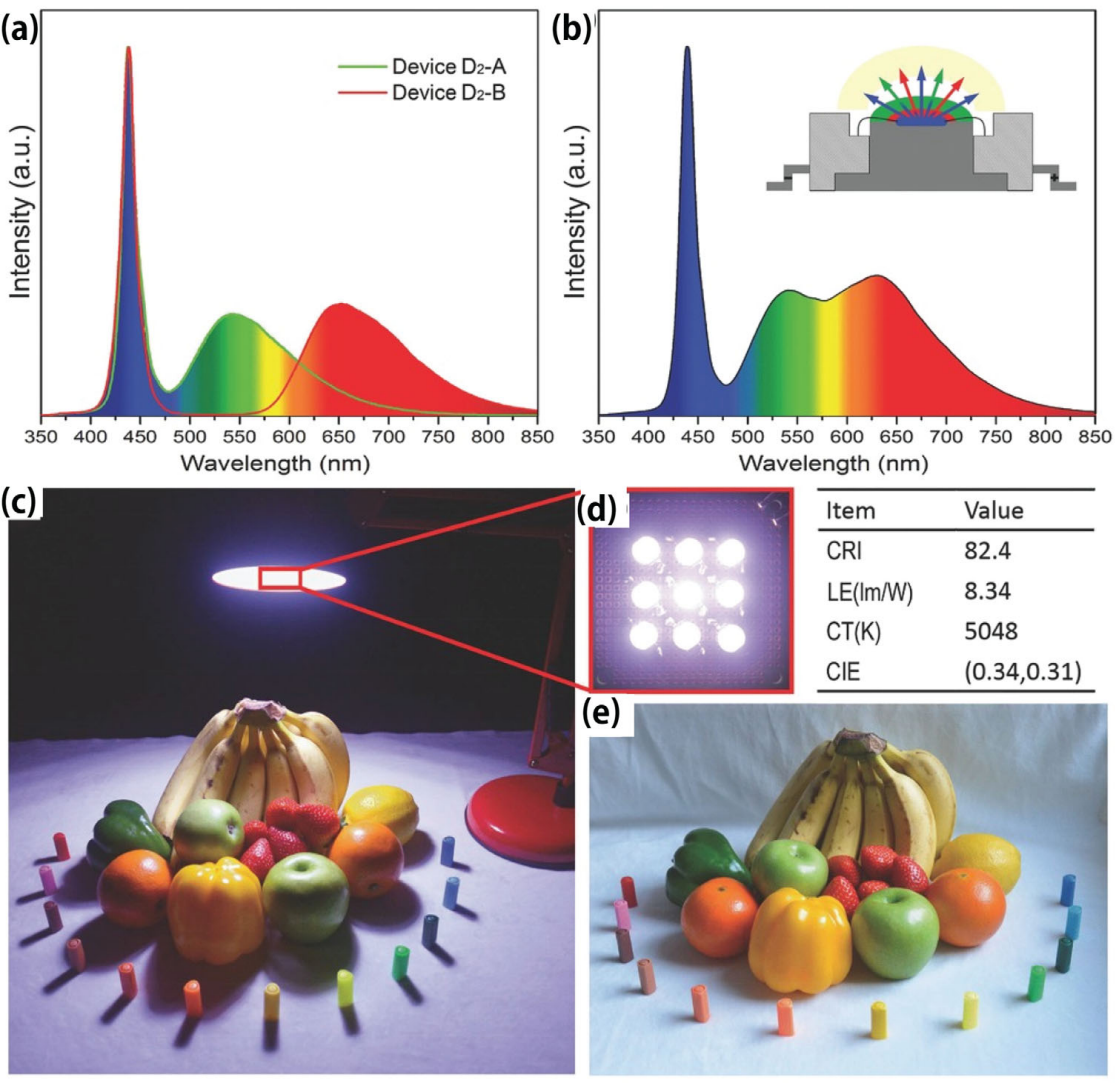
Fluorescent CQDs are applied to light‐emitting devices [120]. Reproduced with permission of Ref. Copyright 2017, John Wiley and Sons.

Wang et al. [121]. synthesized red luminescent CQDs with a quantum yield of 53% using a solvothermal method. Using previously reported green and blue CQDs, three CQDs polymethyl methacrylate films were successfully obtained. This is the first study to construct a warm‐white LED that combines quantum dots with a UV lamp chip. This LED is cost‐effective and environmentally friendly while maintaining high performance. The findings of this study provide a new avenue for the study of warm‐white Led with CQDs. A diagram of the light‐emitting device prepared using the CQDs and its performance is shown in Figure 19.

### 4.4 Photocatalysis

The LCQDS used for photocatalysis have nanoscale carbon cores that can be electronically modified by doped heteroatoms, such as nitrogen, sulfur, or phosphorus, to enhance their photocatalytic activity [122]. The surface of LCQDs is modified by a variety of functional groups, which increases their stability in solvents. Further functionalization pathways are provided through chemical modifications such as the introduction of photosensitive groups or catalytically active sites [123].

The initiation of photocatalysis under illumination is attributed to the photocatalytic process [65]. Under light irradiation, the photocatalytic process involves the initiation of redox reactions to degrade pollutants, purify the environment, and promote material transformations. Ma et al. [124]. prepared LCQDs doped with Ni metal‐organic layers using red phosphorus‐assisted ball milling, which revealed the potential of this novel photocatalyst for tetracycline degradation. Jiang et al. [125]. synthesized LCQDs from lignin extracted from black liquor via nitric acid oxidation, dialysis, and aromatic carbonization. The LCQDs/TiO_2_ photocatalysts were obtained by hydrothermal treatment of the LCQDs with TiO_2_ compounds and reduction with NaBH_4_. These results show that the up‐conversion performance of the LCQDs/TiO_2_ photocatalyst is beneficial for TiO_2_ to generate more photoelectrons. By taking advantage of the broader visible and near‐infrared wavelength ranges of sunlight, the photocatalytic efficiency can be enhanced.

**FIGURE 20:**
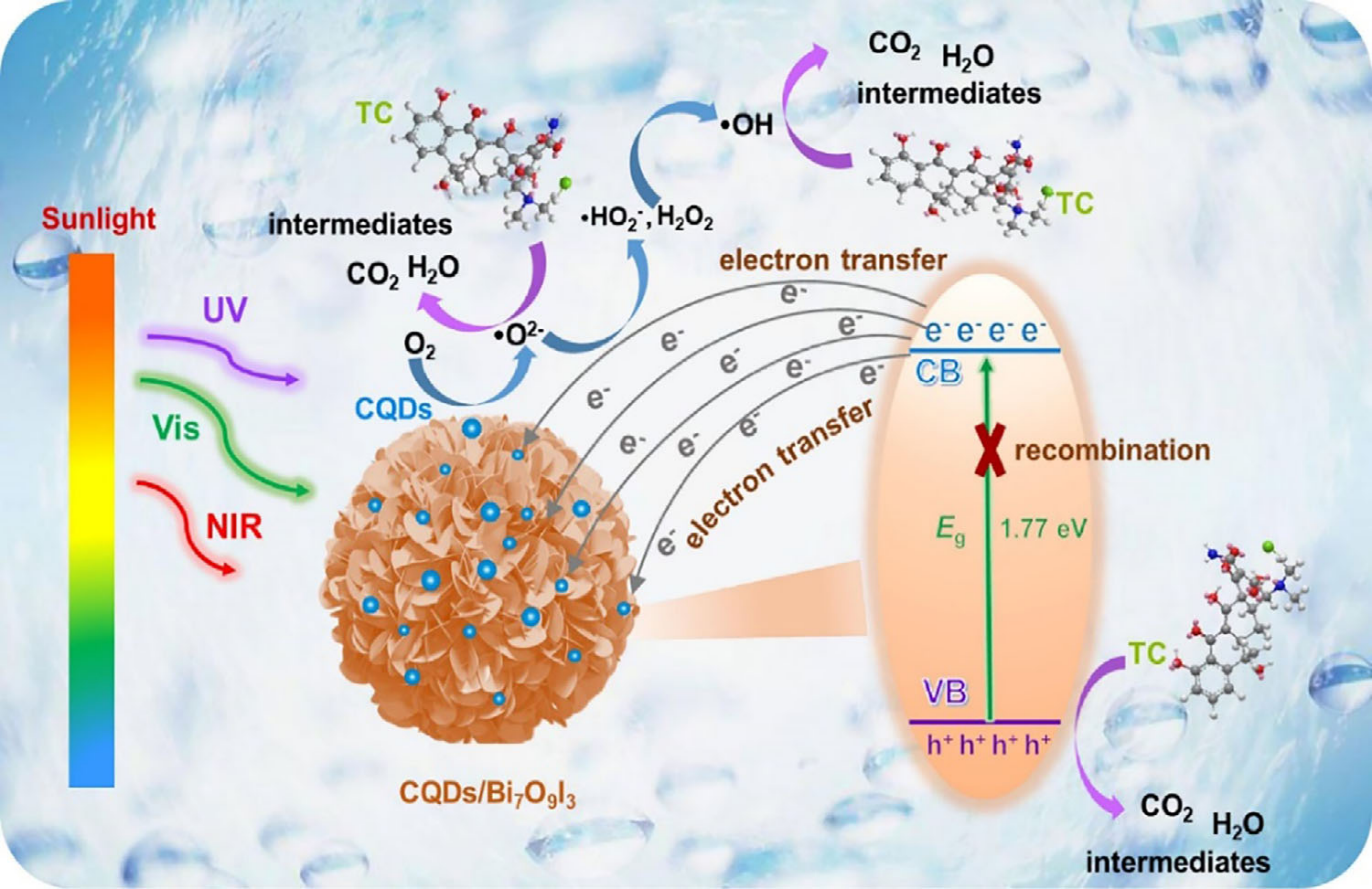
Description of the photocatalysis principle of CQDs/Bi_7_O_9_I_3_ [123]. Reproduced with permission of Ref. Copyright 2023, Elsevier.

Zhang et al. [7]. compounded LCQDs with graphene oxide and evaluated their performance using CV. The results showed good redox performance. It is possible that the combination of GO and LCQDs forms an efficient electron transfer pathway. In addition, LCQDs were uniformly distributed on graphene oxide, providing more active sites. Gao et al. [126]. synthesized CQDs from lignin extracted from poplars and fixed them on TiO_2_ nanosheets. The modified TiO_2_ nanosheets exhibited strong photocatalytic CO_2_ reduction ability. A schematic of the catalytic principle of the CQD composites is shown in Figure 20.

### 4.5 Fluorescent Anti‐Counterfeiting Applications

LCQDs have been found a niche applications in anticounterfeiting owing to their distinctive photoluminescent properties. LCQDs can be integrated into inks and coatings to create security features that are difficult to replicate, thus providing protection against counterfeiting. The fluorescence of LCQDs can be determined by their size, shape, and surface functionalization. Consequently, advanced and safe inks that emit a specific light color under ultraviolet irradiation can be successfully developed [102]. This characteristic can be used to verify branded products, banknotes, and other relevant documents. Furthermore, the sustainability and cost‐effectiveness of LCQDs make them attractive options for large‐scale anticounterfeiting applications [127]. The ability to produce LCQDs with customized lignin characteristics plays an important role in enhancing the security and reliability of various products and documents on the market.

Zhu et al. [128]. synthesized N/S‐doped quantum dots using lignin as the starting material. The prepared CQDs displayed fluorescence in the blue‐to‐yellow‐green range. The fluorescence quantum yield of the N, S‐doped CQDs synthesized by 2, 4‐diamino benzene sulfonic acid was the highest, reaching 30.5%. These yellow‐green luminescent CQDs displayed remarkable characteristics, including bright fluorescence, strong water solubility, and excellent chemical stability, making them suitable for anti‐counterfeit printing applications.

Wang et al. [129]. synthesized graphene quantum dots (GQDs) from lignin via a non‐oxidizing method. The GQDs exhibited concentration‐dependent photoluminescence emissions. Notably, these GQDs demonstrated remarkable ultralong‐term photostability. As a result of these findings, these concentration‐dependent emission GQDs show great potential for a wide range of applications, such as anti‐counterfeiting and bioimaging.

Park et al. [130]. successfully synthesized CQDs from waste paper using a traditional water/solvothermal method. These CQDs exhibit excellent optical properties and chemical and light stabilities. Therefore, anti‐counterfeiting inks and flexible fluorescent films can be easily developed using their unique optical properties and superior chemical and photostabilities.

**FIGURE 21:**
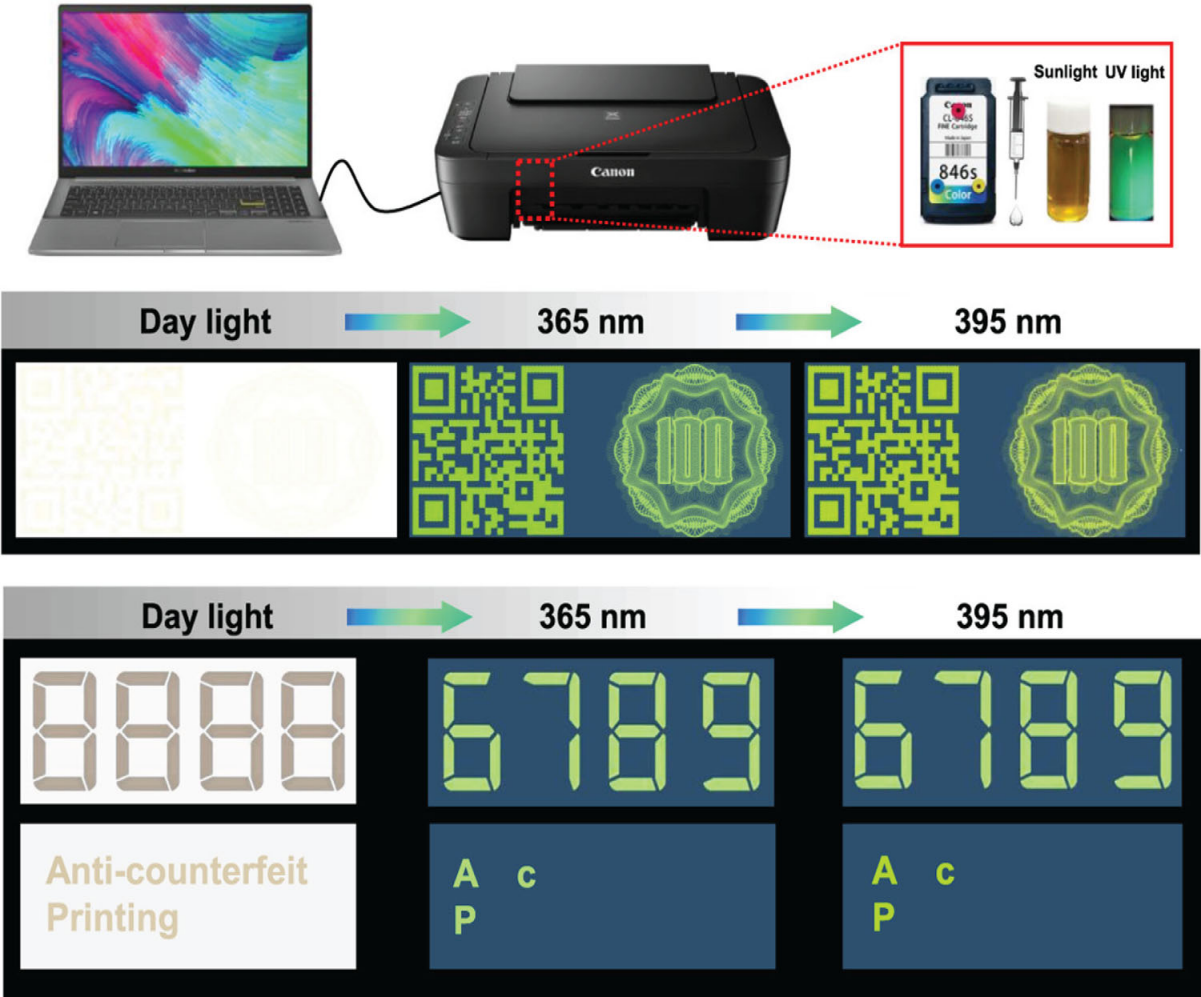
The anti‐counterfeit applications of Y‐CQDs [128]. Reproduced with permission of Ref. Copyright 2021, American Chemical Society.

Wang et al. [131]. successfully synthesized colloidal spheres, demonstrating the pioneering use of lignin as a biophotonic material with customizable structural colors. This breakthrough reveals the enormous potential of lignin resources as environmentally friendly and biocompatible photonic materials for various advanced optical applications, including photonic devices and anti‐counterfeiting labels, thereby establishing the prospect of structural color pigments. In conclusion, LCQDs have the potential to be widely used in the field of anti‐counterfeiting and significantly contribute to social progress. The illustration shows the application of CQDs in anti‐counterfeiting ink and anti‐counterfeiting two‐dimensional code (Figure 21).

## 5 Summary and Outlook

To date, CQDs have displayed tremendous potential in various applications, such as photocatalysis, fluorescent probes, bioimaging, luminescent devices, and anti‐counterfeiting.

Different methods for preparing LCQDs have their advantages and disadvantages. For example, HTC can be used to synthesize LCQDs with good size and dispersion uniformity at lower temperatures and pressures, but with long reaction times and low product purity. The microwave method has the advantages of fast reaction, high purity, and fast synthesis. However, this method has higher equipment requirements and high production costs. The ultrasonic method has the characteristics of simple operation and low cost and can effectively prepare small LCQDs. However, this method produces byproducts, resulting in a lower purity of the prepared product. The chemical oxidation method can introduce more functional groups on the surface of LCQDs, which expands their application potential. The size and appearance of the LCQDs can be adjusted by changing the oxidation conditions. However, this method is expensive and requires complex postprocessing. High‐quality LCQDs with uniform sizes and regular shapes can be prepared in large quantities by laser etching, and the preparation process is environmentally friendly. However, the disadvantages of this method include high equipment costs and high operational technology requirements. By adjusting the electrochemical oxidation parameters, the size and dispersion of the CQDs can be precisely controlled by electrochemical oxidation, and the microstructure of the CQDs can be changed to a certain extent. This method has a low production cost and high product purity; however, it requires a long reaction time, and it is difficult to achieve mass production. Therefore, researchers can choose an appropriate method to prepare LCQDS according to their specific requirements. Based on this premise, researchers can select appropriate methods for preparing quantum dots according to specific requirements.

Owing to their unique physical and chemical properties, LCQDs have a wide range of potential applications in various fields. Some of the most promising applications are bioimaging and biosensing. The biocompatibility, low toxicity, and tunable optical properties of LCQDs render them ideal materials for biomedical applications. They can be used as fluorescent markers for cell imaging, which helps scientists observe the internal structures and biological processes of cells in greater detail. In addition, CQDs can be used as drug carriers to improve the efficacy of drugs and reduce side effects, owing to the adsorption and controlled release characteristics of drugs with rich surface functional groups. This multifunctionality, coupled with the environmentally friendly nature derived from lignin, a renewable resource, suggests a promising future for LCQDs in the biomedical sector.

Lignin, a natural biomass resource with extensive renewability, rich benzene ring structure, easy modification capability, and good molecular self‐assembly performance, has attracted increasing attention for CQDs preparation and utilization. Currently, hydrothermal and thermal decomposition methods are primarily used for the synthesis of lignin‐derived CQDs. Hydrothermal methods can be used to prepare high‐quality CQDs, including LCQDs, at low temperatures. Furthermore, self‐assembly and surface modification techniques allow adjustment of LCQDs' surface properties, morphology, fluorescence characteristics, and stability of LCQDs. Altering the surface functional groups of the CQDs can result in different surface modification effects. In summary, the lignin‐based synthesis is an environmentally friendly and efficient method for producing CQDs. Additionally, owing to their natural renewability and biocompatibility, LCQDs have tremendous potential in various fields, such as biology, pharmaceuticals, catalysis, and anti‐counterfeiting.

Realizing the industrial application of CQDs is a lengthy process. Although the development and utilization of LCQDs are gradually accelerating, their overall applications remain relatively limited. It remains a great challenge to achieve a high yield of CQDs, effectively solve the solid‐state cracking phenomenon, and improve the performance of CQDs in various applications. The goal of researchers is to successfully solve problems related to the high‐value‐added utilization of lignin.

## Author Contributions

Conceptualization, Maoqing Fu and Tianci Qin; methodology, Fengfeng Li and Zhili Zhang; validation, Tianci Qin, Fengfeng Li, and Xiuxin Yin; formal analysis, Zhili Zhang and Maoqing Fu; investigation, Maoqing Fu; resources, Shaolong Sun; data curation, Shaolong Sun and Fengfeng Li; writing‐original draft preparation, Zhili Zhang and Xiuxin Yin; writing‐review and editing, Xiuxin Yin and Zhili Zhang; visualization, Zhili Zhang and Xiuxin Yin; supervision, Xingxiang Ji and Yuanyuan Wang; project administration, Xingxiang Ji and Shaolong Sun; funding acquisition, Fengfeng Li and Shaolong Sun. All authors have read and agreed to the published version of the manuscript.

## Conflicts of Interest

The authors declare no conflicts of interest.

## Data Availability

Data are contained within this article.
